# Supplementary material for: Evaluating the structure-based virtual screening performance of SARS-CoV-2 main protease: A benchmarking approach and a multistage screening example against the wild-type and Omicron variants
Source: PLoS One. 2025 Feb 19;20(2):e0318712. doi: 10.1371/journal.pone.0318712 (PMC11838920; doi:10.1371/journal.pone.0318712)
Supplement: S1 File — It also contains Table A for the active set chemotype clusters used for benchmarking, and Table B for the active set compounds used for benchmarking. It includes Table C showing the structures of Table 1. SI_file02 contains the SMILES of the active and decoy molecules.(ZIP) [file pone.0318712.s001.zip › SI_file01_02/SI_file01.docx]

**Supporting Information**

Evaluating the structure-based virtual screening performance of SARS-CoV-2 main protease: A benchmarking approach and a multistage screening example against the wild-type and Omicron variants

Noha Galal^1,2^, Botros Y. Beshay^3^, Omar Soliman^4^, Muhammad I. Ismail^5^, Mohamed Abdelfadil^6^, Mohamed El-Hadidi^7,8^, Reem K. Arafa^1,2*¶^, Tamer M. Ibrahim^6,8*¶^

^1^Drug Design and Discovery Laboratory, Zewail City of Science and Technology, October Gardens, 6^th^ of October City, Giza, Egypt

^2^Biomedical Sciences Program, UST, Zewail City of Science and Technology, October Gardens, 6^th^ of October City, Giza, Egypt

^3^Pharmaceutical Chemistry Department (Pharmaceutical Sciences Division), College of Pharmacy, Arab Academy for Science, Technology and Maritime Transport, Alexandria, Egypt

^4^Genomics Program, Children's Cancer Hospital Egypt, Cairo, Egypt.

^5^Department of Pharmaceutical Chemistry, Faculty of Pharmacy, The British University in Egypt, Al-Sherouk City, Cairo-Suez Desert Road, Cairo, Egypt

^6^Department of Pharmaceutical Chemistry, Faculty of Pharmacy, Kafrelsheikh University, Kafrelsheikh, Egypt

^7^Institute of Cancer and Genomic Sciences (ICGS), School of Medical Sciences, College of Medicine and Health, University of Birmingham Dubai, Dubai, United Arab Emirates

^8^Center for Informatics Science (CIS), School of Information Technology and Computer Science (ITCS), Nile University, Giza, Egypt

^*^ Corresponding author

E-mail: [rkhidr@zewailcity.edu.eg](mailto:rkhidr@zewailcity.edu.eg) (RKA)

E-mail: [Tamer_Mohamad@pharm.kfs.edu.eg](mailto:Tamer_Mohamad@pharm.kfs.edu.eg) (TMI)

^¶^ These authors contributed equally to this work

## Methods

### Preparation of small molecules

The “Molecule wash” module yielded reliable protonation states by protonation of strong bases and deprotonation of strong acids (if required). For each compound, one protonation state was produced at pH 7.0. Minimization of the energy carried by the Amber: 10EHT force field at a gradient of 0.01 RMSD. At the same time, the remaining options were kept at default settings. Furthermore, one conformer was saved. The prepared compounds were saved as SD files and utilized in docking studies with FRED.

### Preparation of the protein structures

In our docking study, we removed water molecules after removing any unnecessary ions, redundant chains, crystallization molecules, and unessential solvent molecules (if any), MOE “Quickprep” function was used with default settings. Such parameters incorporate using the “Protonate 3D” function to improve the H-bonding network and allow ASN/GLN/HIS to flip for optimal protonation and H-bonding networking. Furthermore, the ligand and binding site atoms were optimized by reducing the energy to an RMS gradient of 0.1 kcal/mol/A, while the constraints of the binding site atoms were imposed using a force constant (strength = 10). The remaining receptor atoms, which are present outside the binding pocket, were kept the same.

### Molecular dynamics simulations

Then, the system was neutralized using NaCl ions with ionic strength of 0.1M concentration. For system energy minimization, the steepest descent minimization algorithm was utilized by a convergence set at 10 kJ/mol and 50,000 steps. At 300 K temperature and 1 atm pressure, each NVT followed by NPT equilibration was conducted for 500 ps. After that, a production run at NPT ensemble was performed for 100 ns. For each equilibration run, temperature coupling was carried out using the V-rescale modified Berendsen thermostat, for equilibration and production runs, a 2 ps time constant Berendsen coupling was employed for pressure coupling[1, 2]. Furthermore, for pressure coupling, the Parrinello-Rahman pressure coupling scheme was utilized for the production runs[3]. Using the Verlet cutoff-scheme with 1.2 cutoff and 1.0 nm switch list distances was for Van der Waals calculations and searching for adjacent atoms. The method of Particle Mesh Ewald was employed for the long-range electrostatics’ calculations within 1.2 nm[4]. The bond lengths were constrained using the LINear Constraint Solver (LINCS) algorithm[5]. The protein molecules’ topology and parameters were generated by applying the CHARMM36 all-atom force field, while the ligand parameters were generated using the SwissParam server[6, 7].

**Table A.** Active set chemotype clusters used for benchmarking.

| **Cluster #** | **Scaffold name** |
| --- | --- |
| **1** | 3,5-diphenyl-2H-[1,3'-bipyridine]-2-one |
| **2** | N-(2-benzyl phenyl)pyrimidine-4-carboxamide |
| **3** | 2-((5-phenyl-[1,2,4]triazolo[1,5-a]pyrimidin-7-yl)thio)-5-(thiophen-2-yl)-1,3,4-oxadiazole |
| **4** | N-(isoquinolin-4-yl)-3,4-dihydronaphthalene-1-carboxamide |
| **5** | N-(isoquinolin-4-yl)-2-(pyridin-3-yl)acetamide |
| **6** | 1-(isoquinolin-4-yl)-3-phenylurea |
| **7** | N-benzyl-2-(isoquinolin-4-yl)-N-(pyridin-2-yl)acetamide |
| **8** | N-(1H-indol-5-yl)furan-2-carboxamide |
| **9** | 2-(phenylamino)-6H-1,3-oxazin-6-one |
| **10** | 2-(3-(4-methylpiperazin-1-yl)phenyl)-N-(4,5-dihydropyridin-3-yl)acetamide |
| **11** | 3-benzyl-7-(pyridin-2-ylthio)-3H-[1,2,3]triazolo[4,5-d]pyrimidine |
| **12** | indolizin-3-yl(phenyl)methanone |
| **13** | N-benzyl-4-(indolin-1-ylmethyl)piperidine-1-carboxamide |
| **14** | 2-phenyl-5-(thiazol-2-ylthio)-1,3,4-oxadiazole |
| **15** | N-(6-phenylimidazo[2,1-b]thiazol-5-yl)benzamide |
| **16** | N-(benzo[d][1,3]dioxol-5-ylmethyl)pyrimidine-4-carboxamide |
| **17** | 8-(thiophene-2-carbonyl)-1,3,8-triazaspiro[4.5]decane-2,4-dione |
| **18** | 2-phenyl-4H-chromen-4-one |
| **19** | isoquinolin-4-yl(4-phenylpiperazin-1-yl)methanone |

**Table B.** The active set compounds used for the benchmarking

| **Serial** | **Structure** | **IC_50_ (µM)** | | **Cluster** | **M.Weight** | **Reference** |
| --- | --- | --- | --- | --- | --- | --- |
|  | 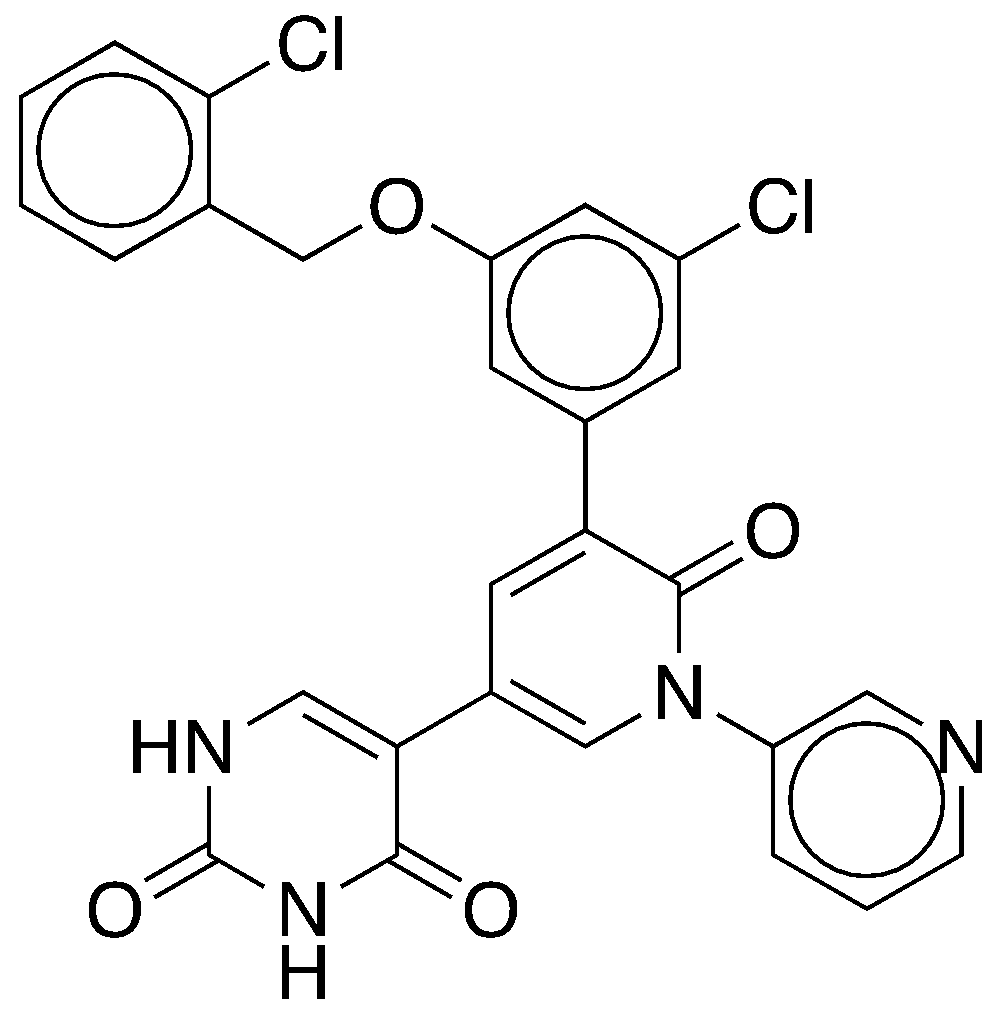 | 0.017 | | 1 | 533.37 | [8] |
|  | 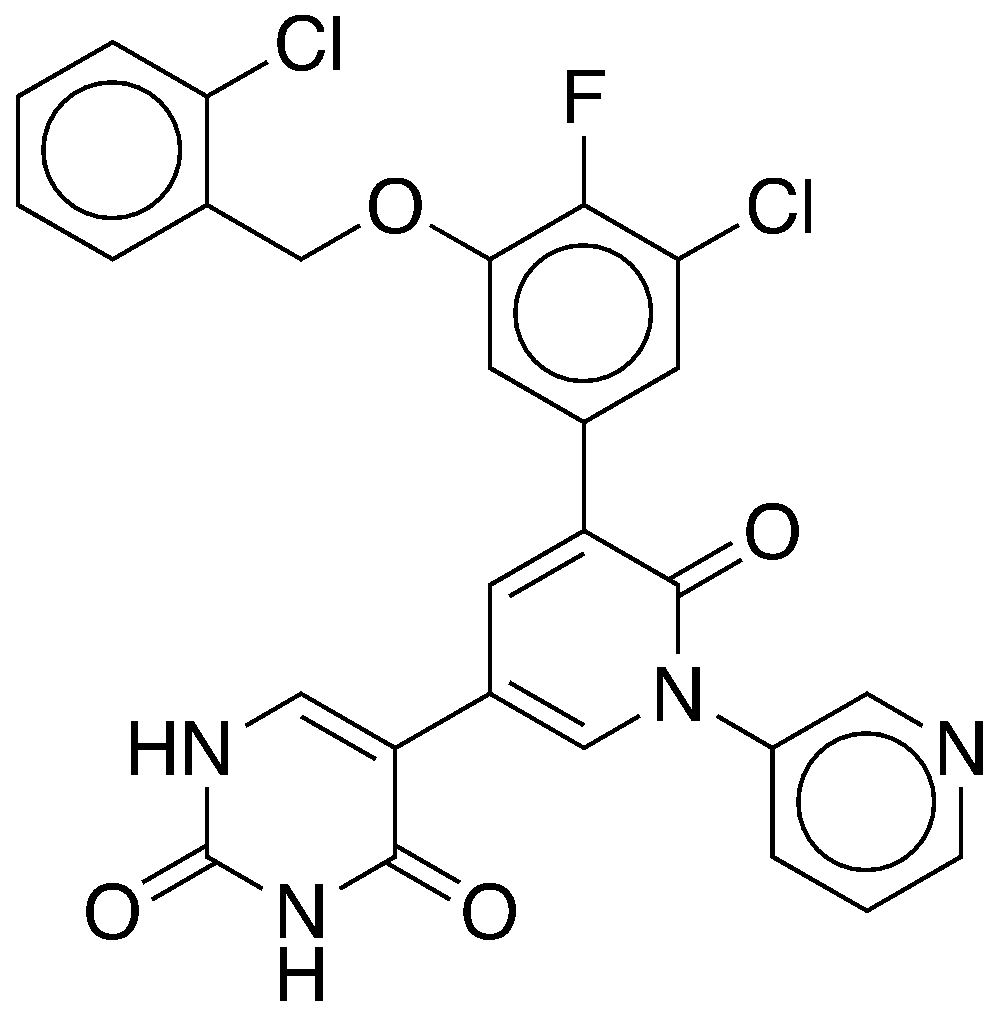 | 0.02 | | 1 | 551.36 | [8] |
|  | 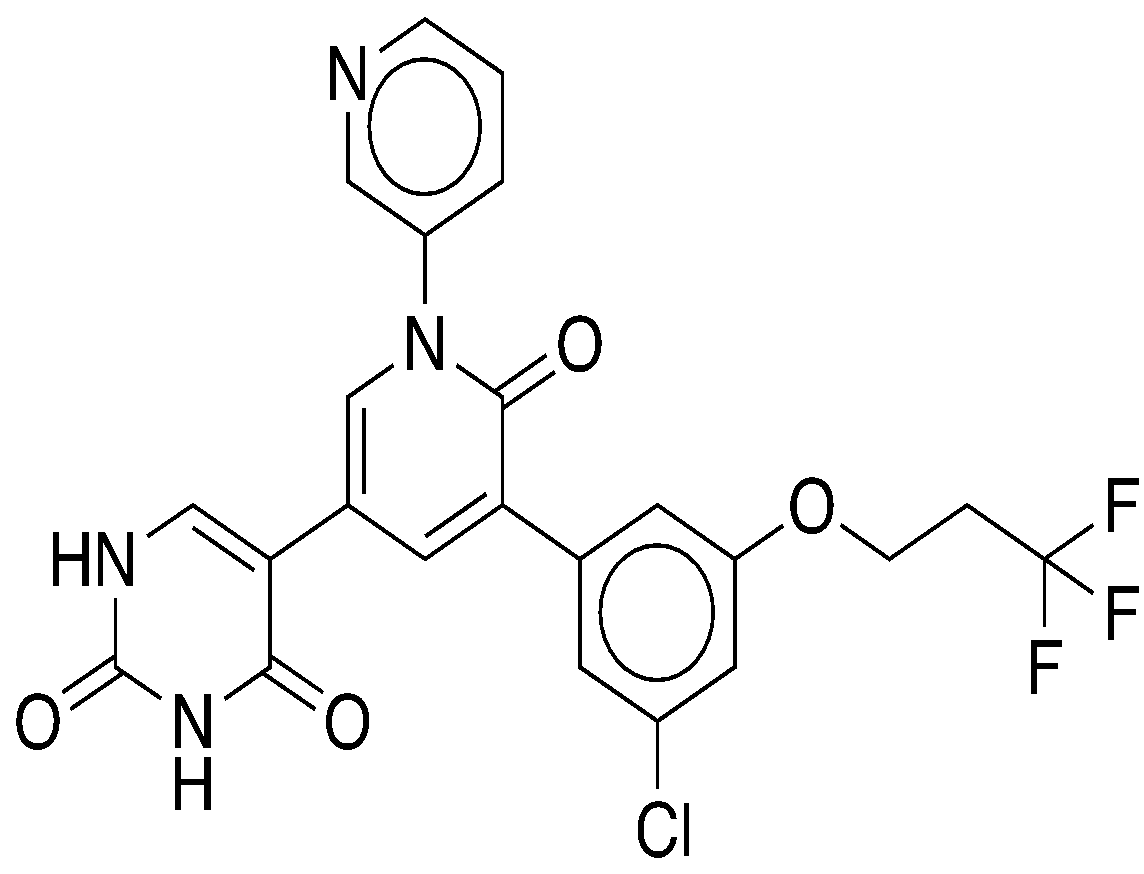 | 0.025 | | 1 | 504.851 | [8] |
|  | 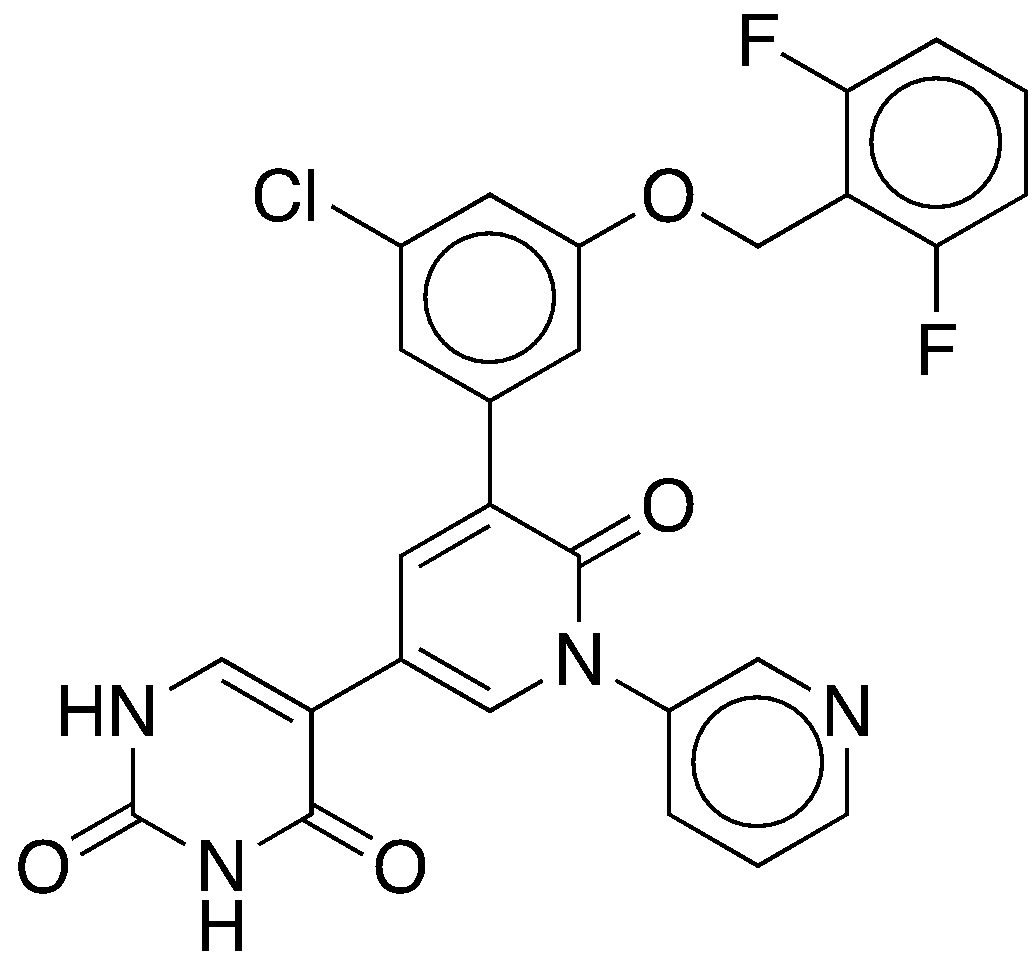 | 0.035 | |  | 534.905 | [8] |
|  | 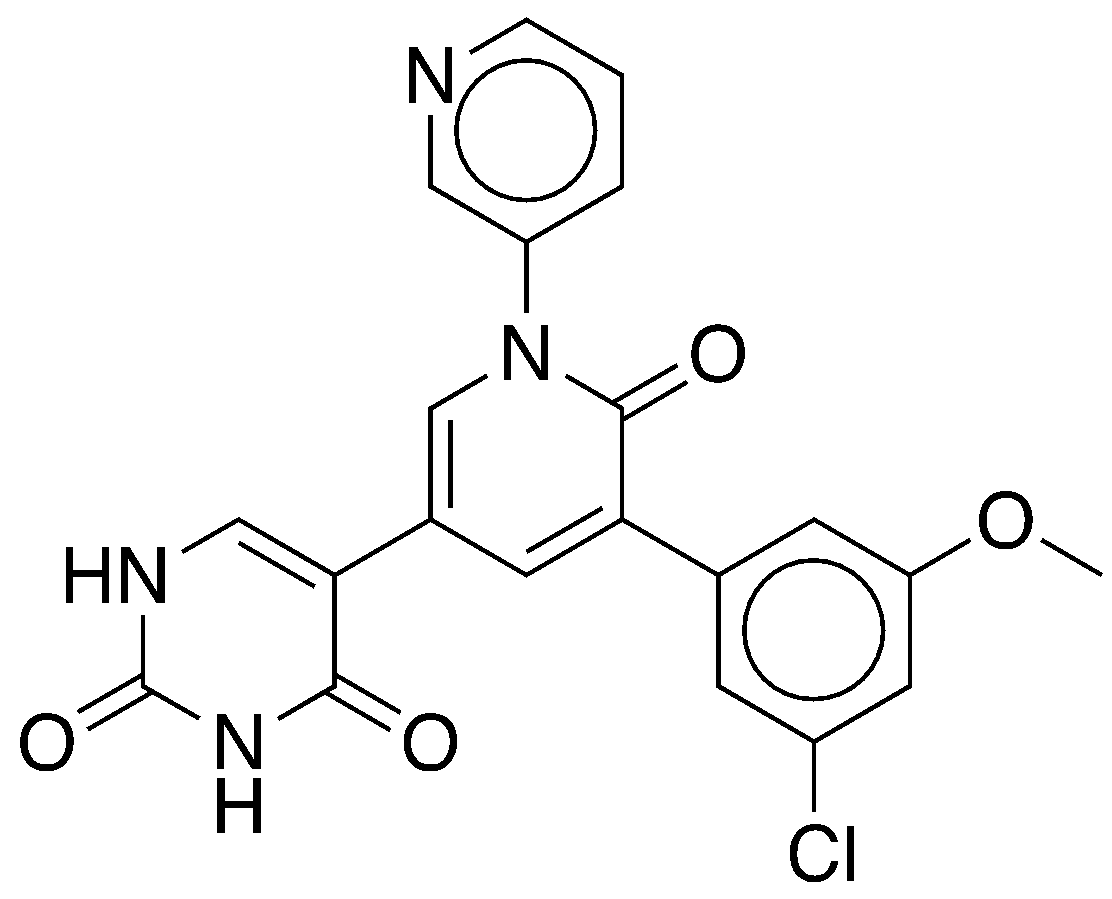 | 1.2 | | 1 | 422.827 | [8] |
|  | 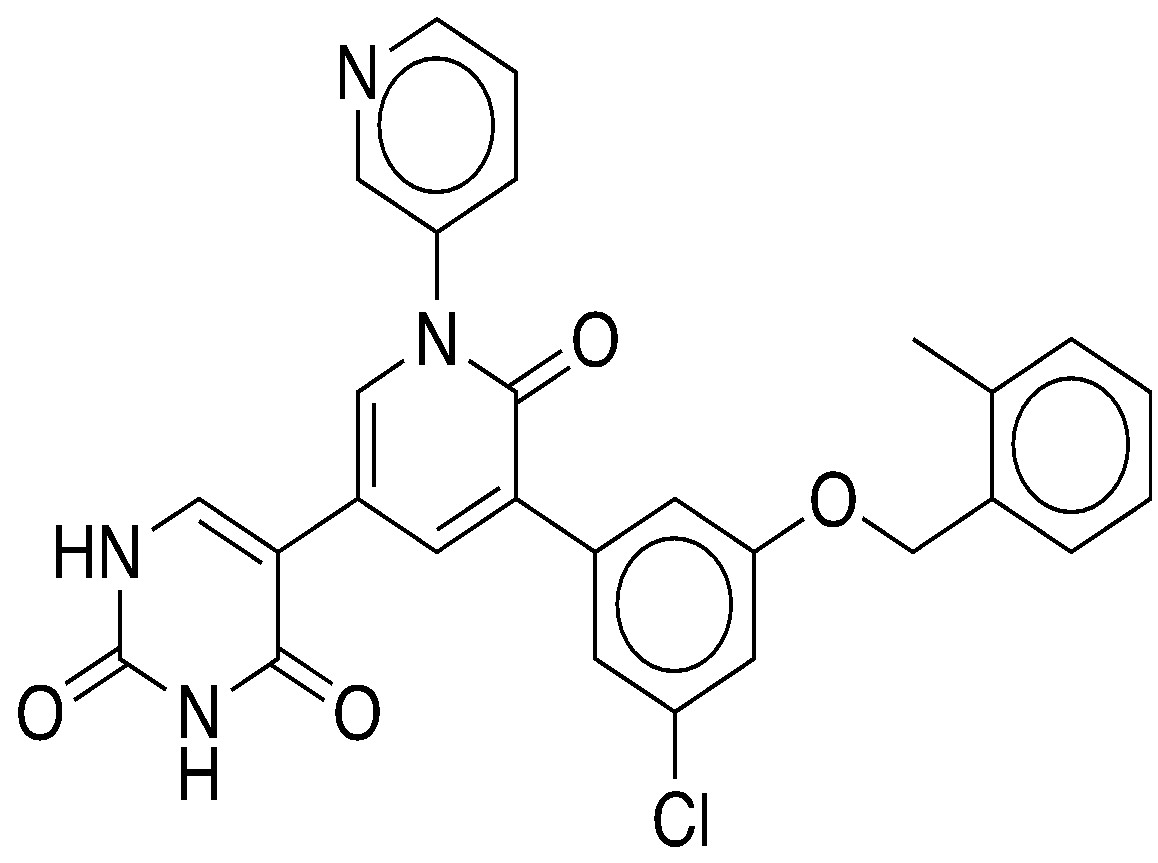 | 0.1 | | 1 | 512.952 | [8] |
|  | 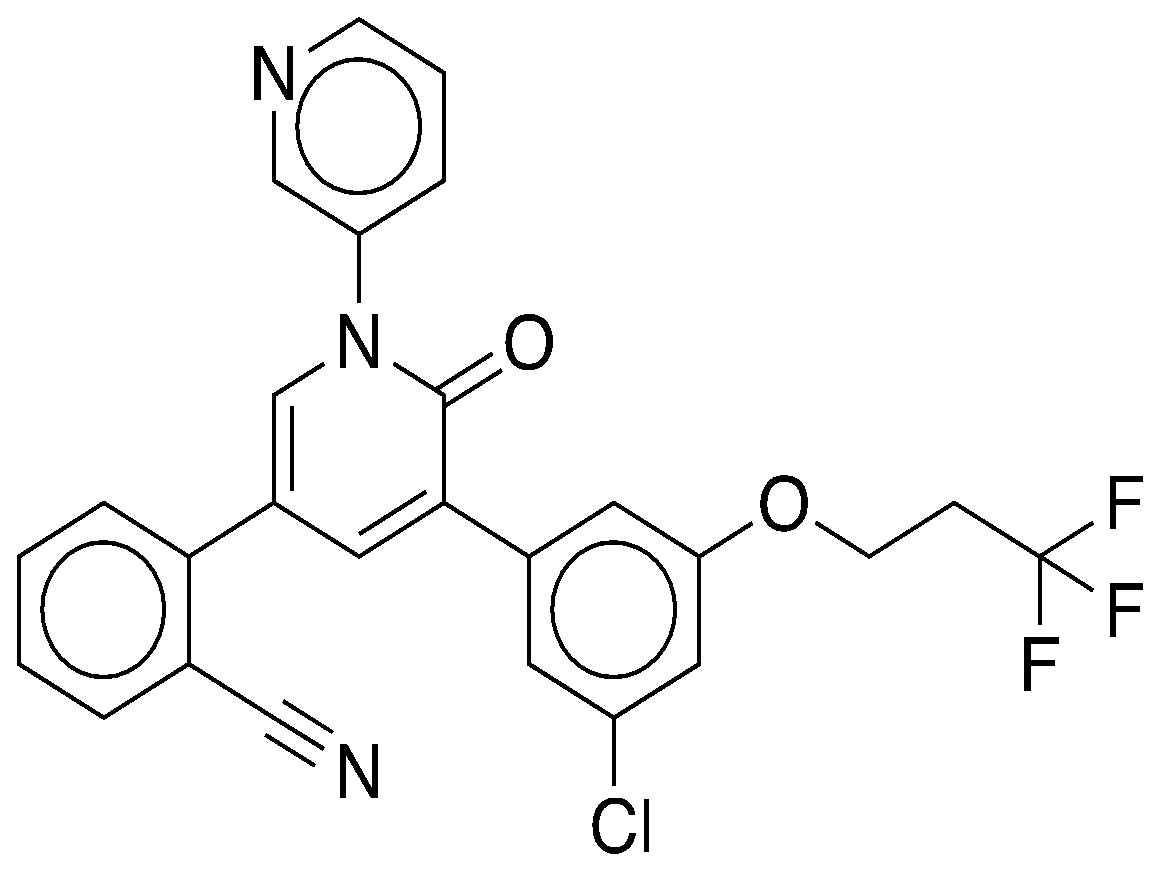 | 0.12 | | 1 | 495.887 | [8] |
|  | 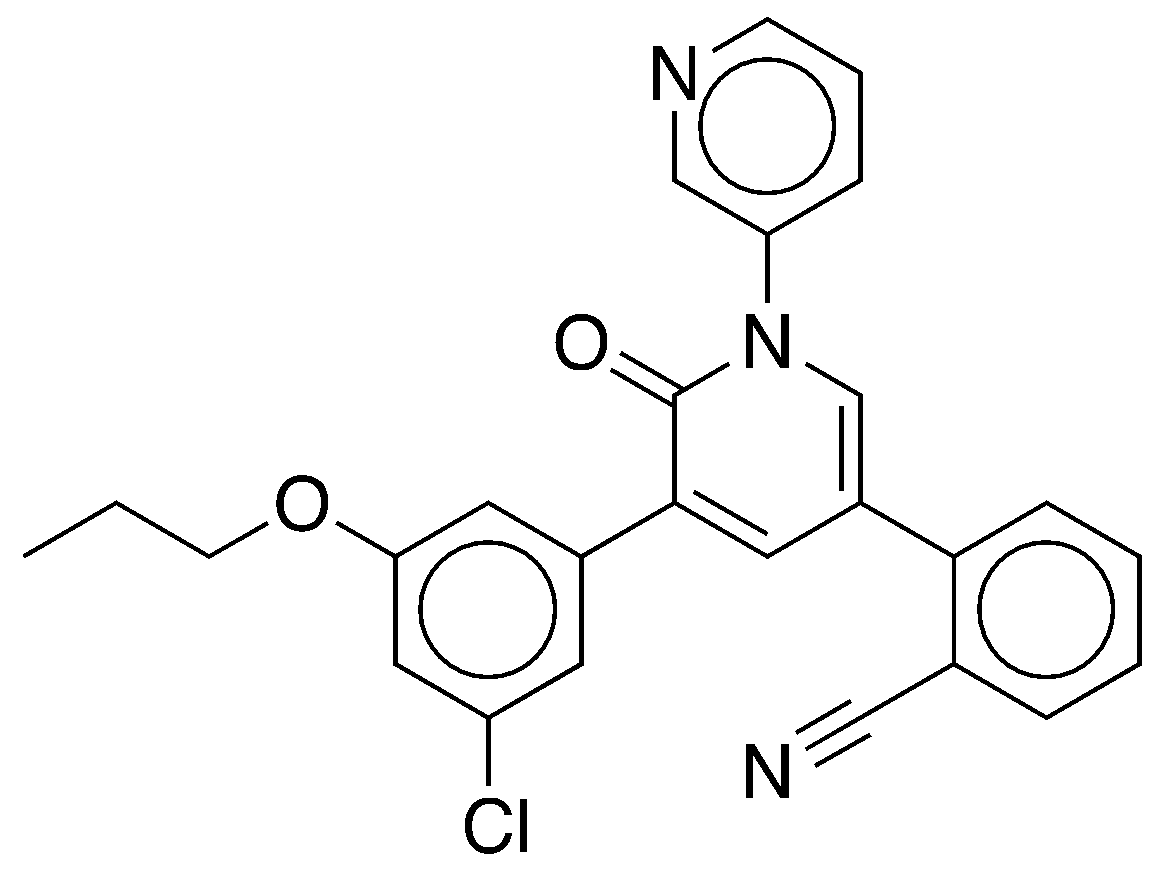 | 0.14 | | 1 | 441.917 | [8] |
|  | 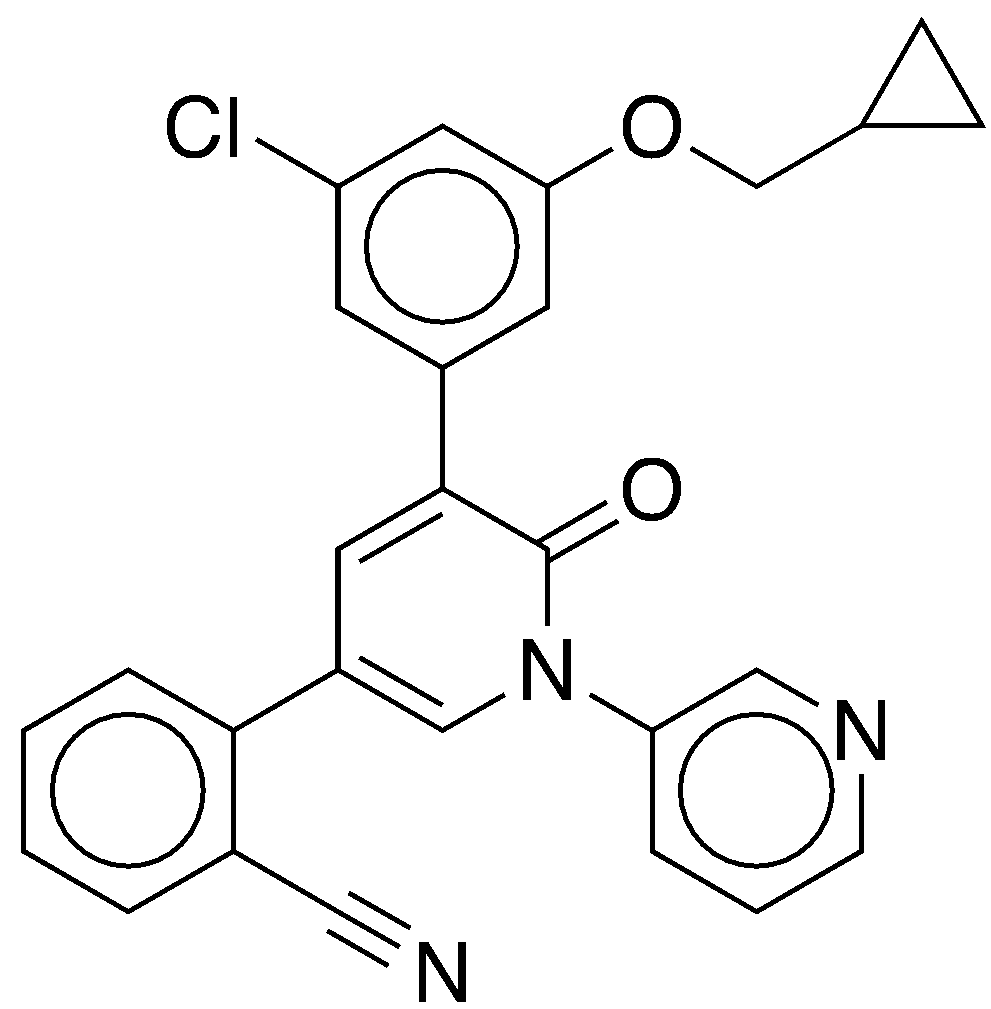 | 0.17 | | 1 | 453.928 | [8] |
|  | 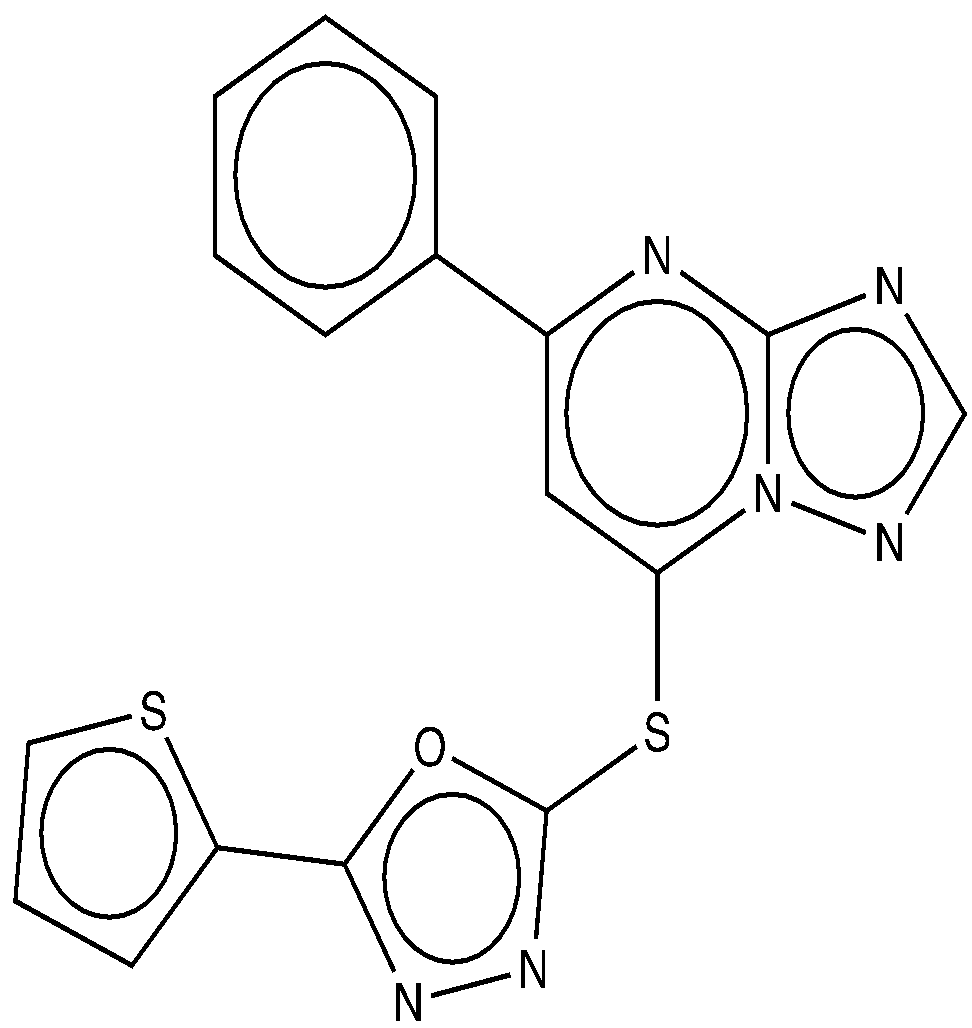 | 0. 22 | | 3 | 378.439 | COVID Moonshot |
|  | 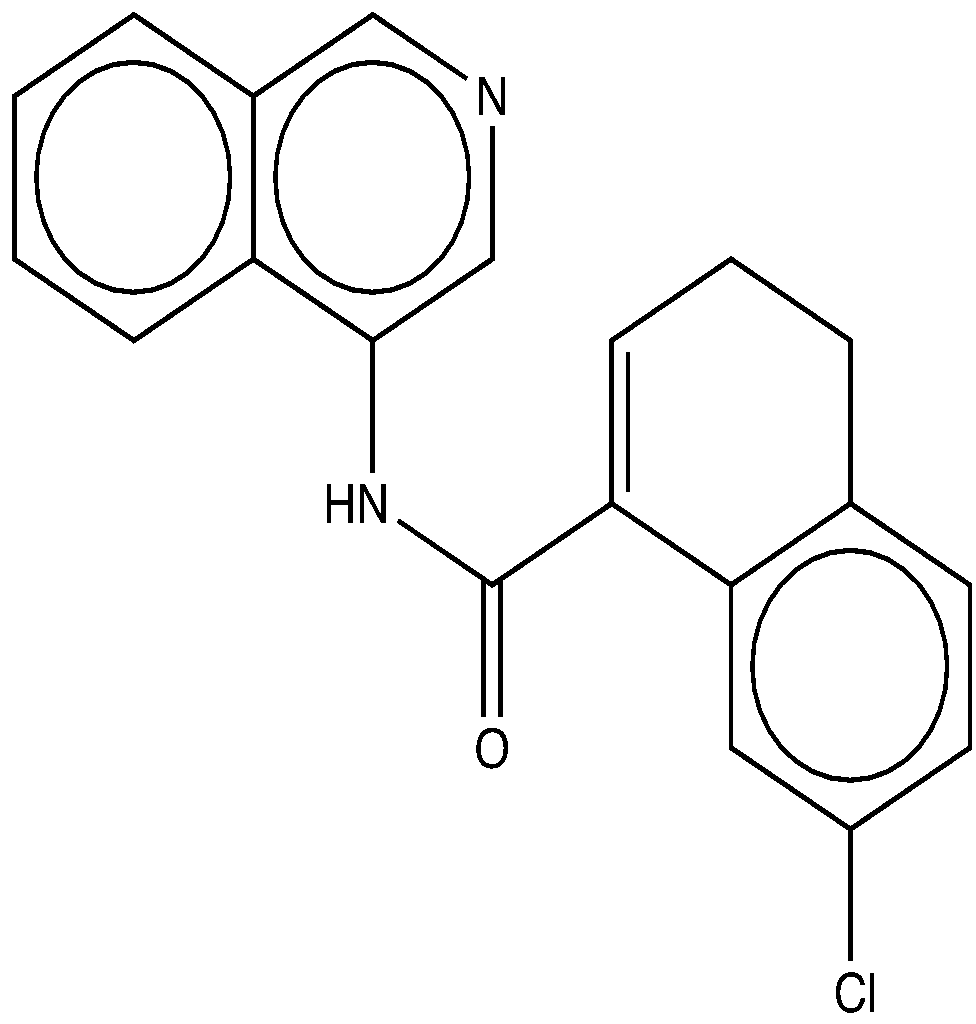 | 0.23 | | 4 | 334.805 | COVID Moonshot |
|  | 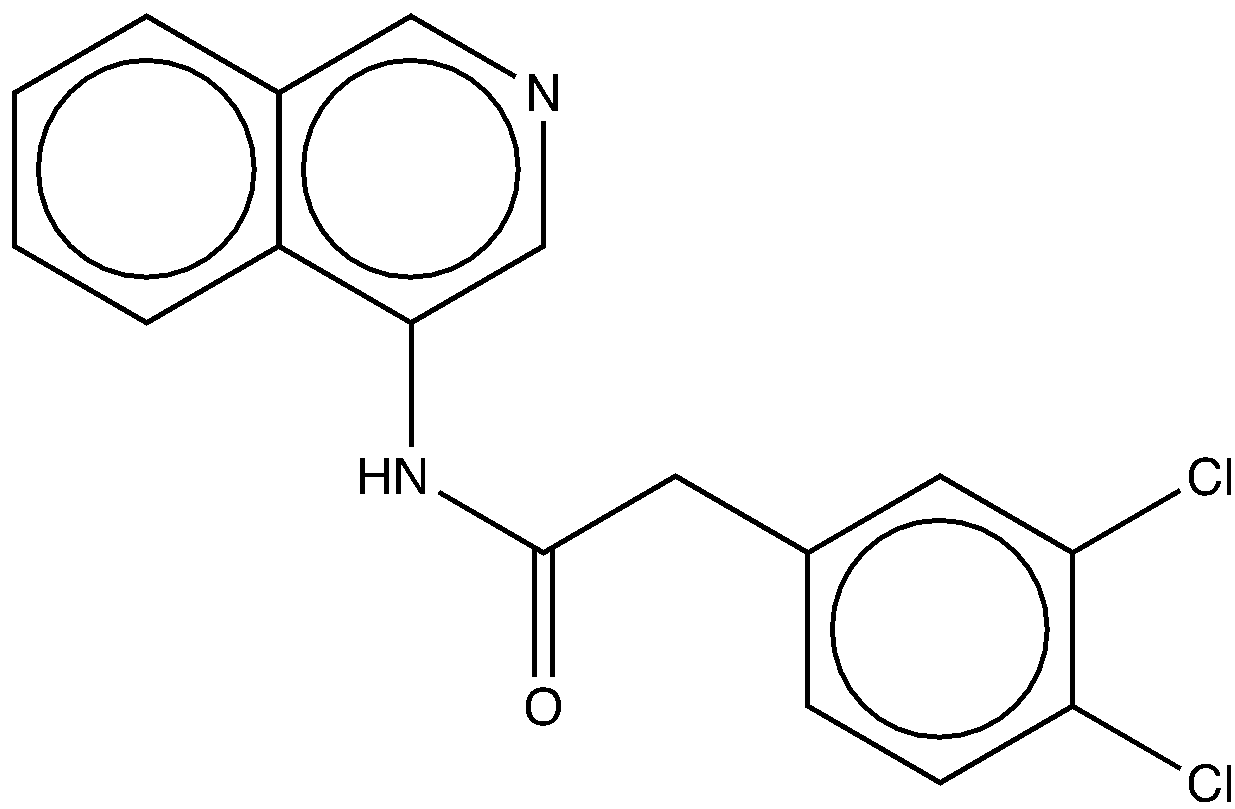 | 0.25 | | 5 | 331.201 | COVID Moonshot |
|  | 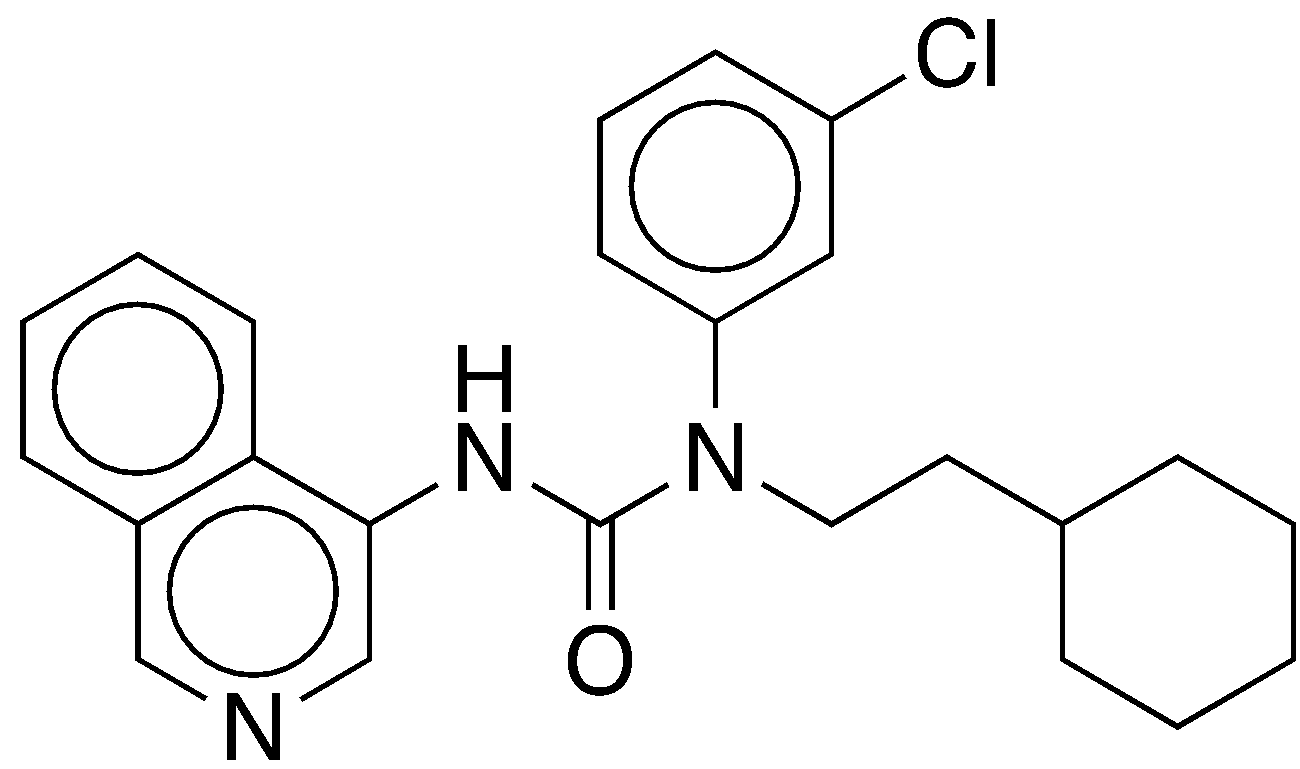 | 0.25 | | 6 | 407.943 | COVID Moonshot |
|  | 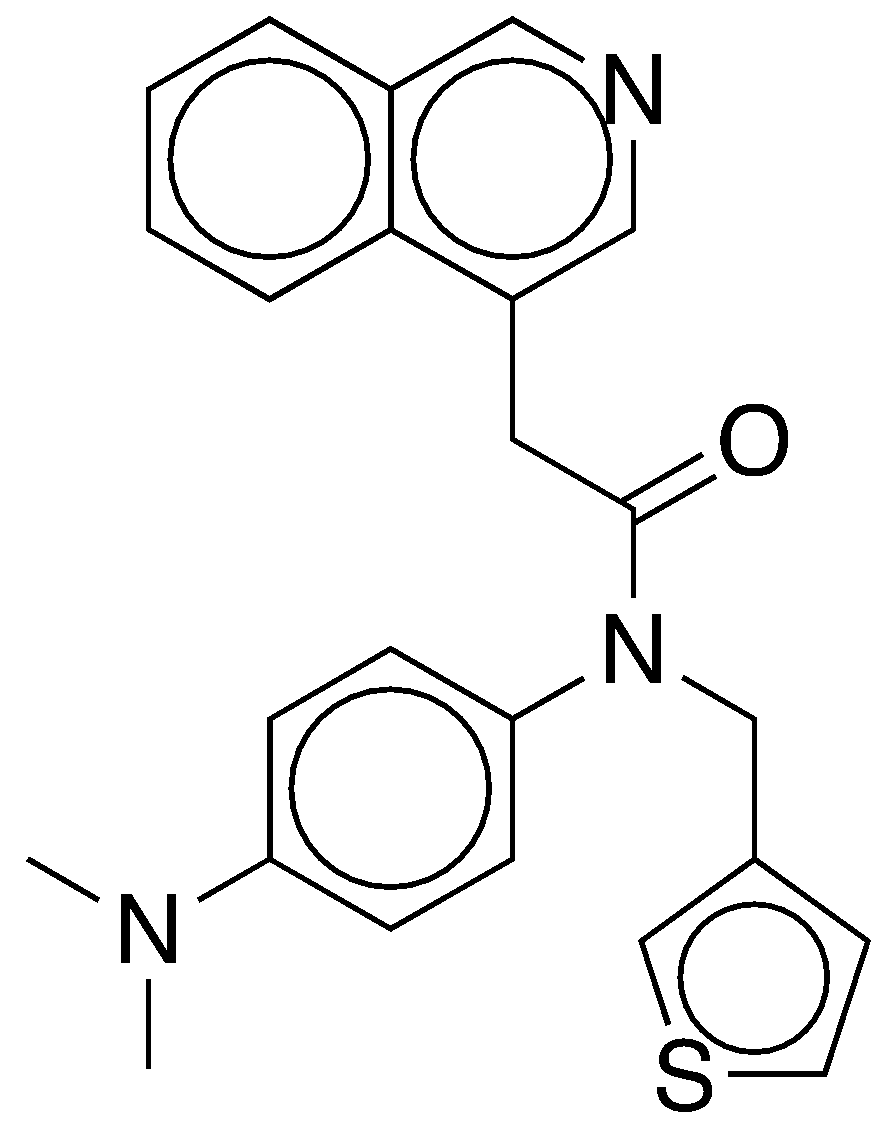 | | 0.28 | 7 | 401.533 | COVID Moonshot |
|  | 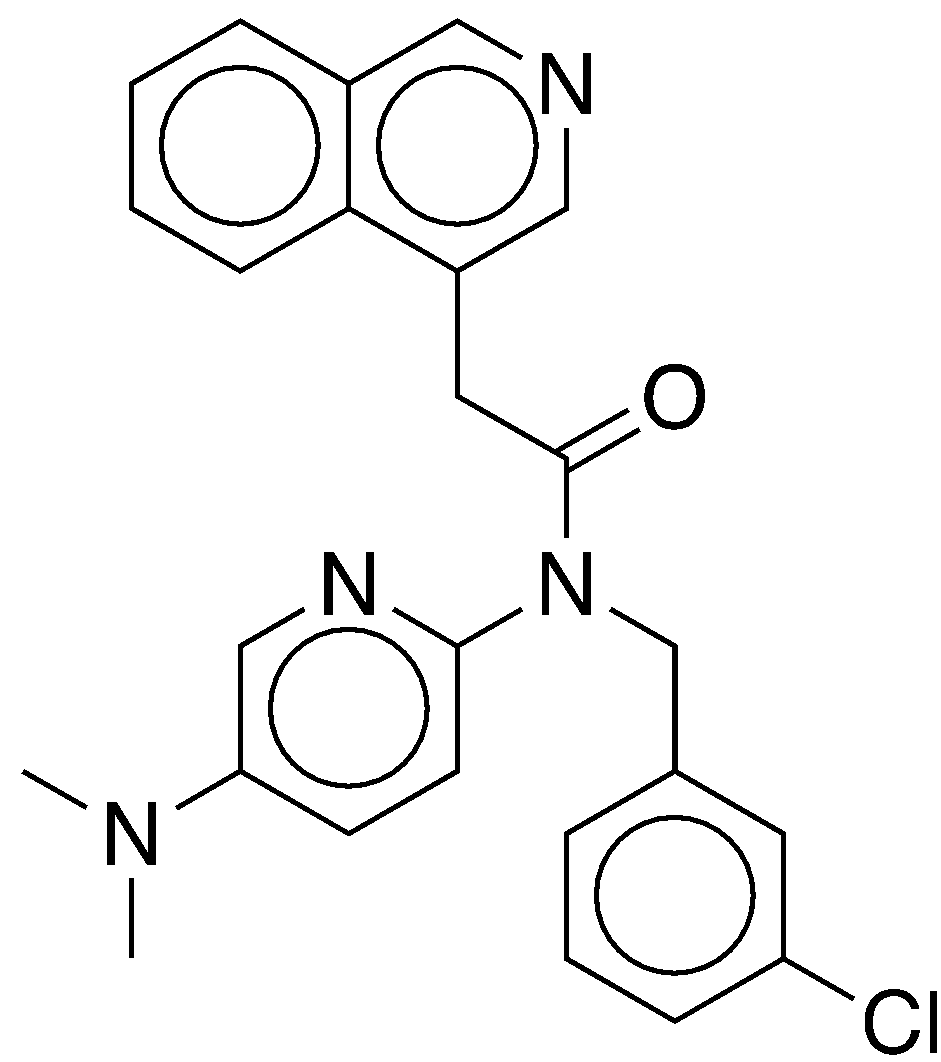 | | 0.28 | 7 | 430.938 | COVID Moonshot |
|  | 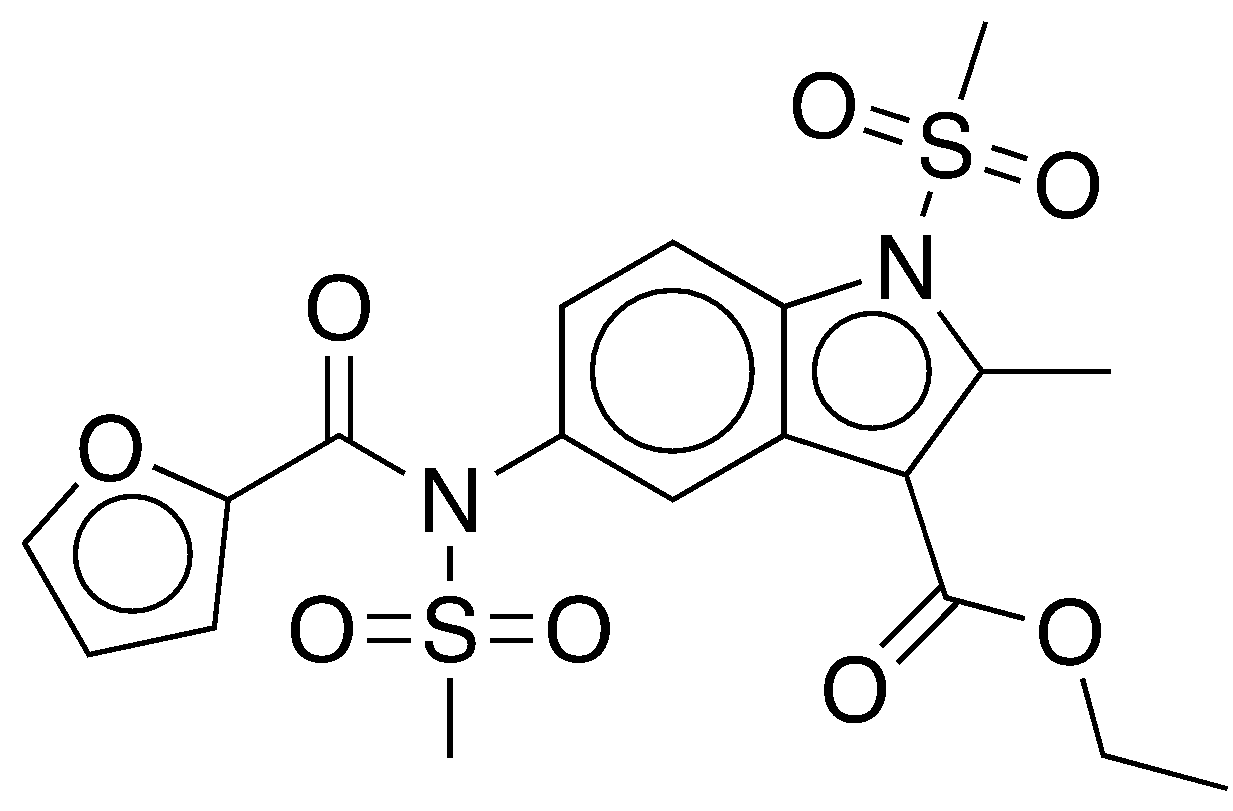 | | 0.28 | 8 | 468.506 | COVID Moonshot |
|  | 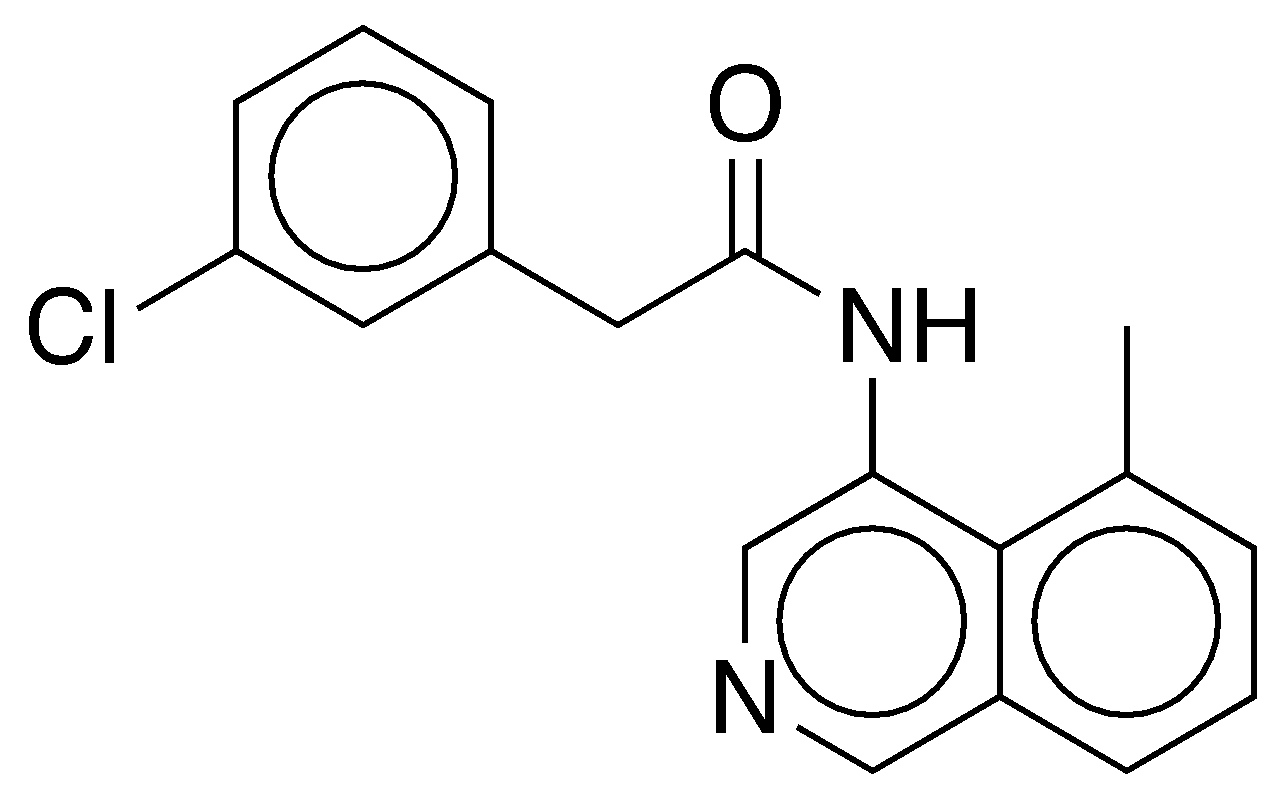 | | 0.33 | 5 | 310.783 | COVID Moonshot |
|  | 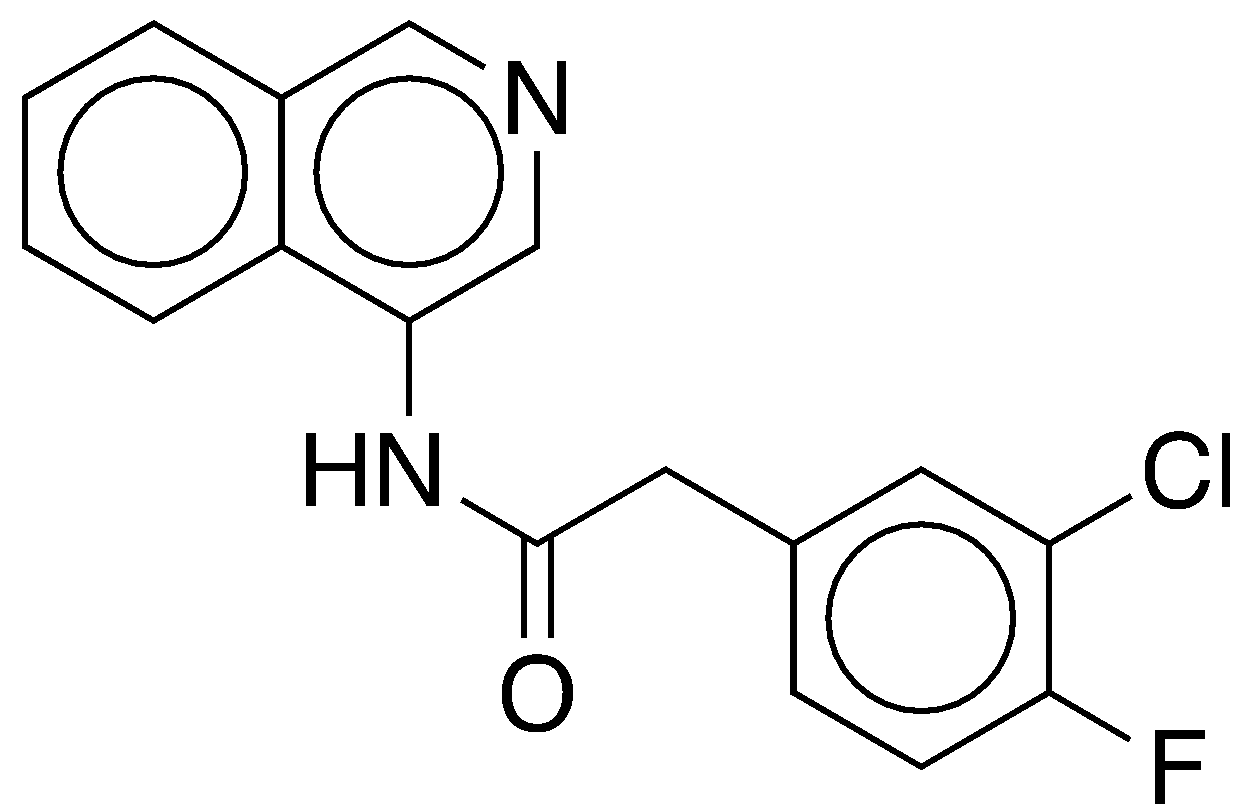 | | 0.37 | 5 | 314.746 | COVID Moonshot |
|  | 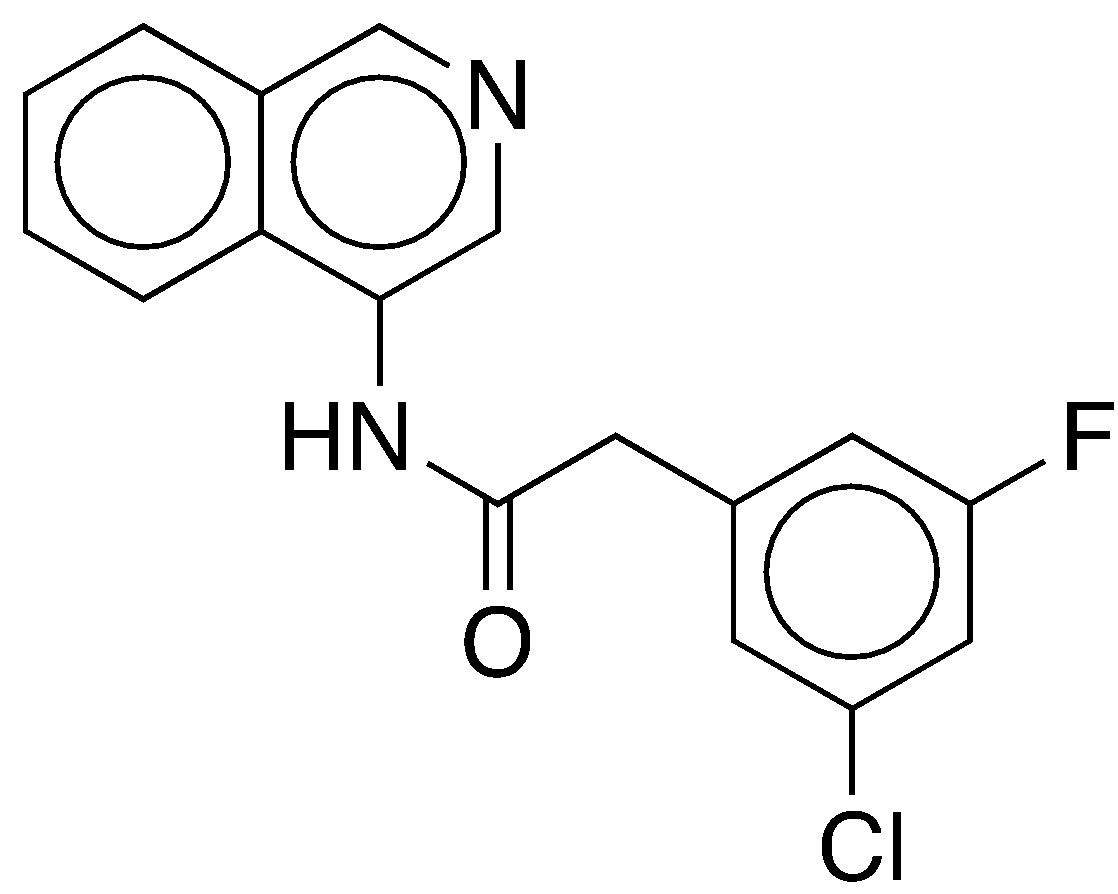 | | 0.38 | 5 | 314.746 | COVID Moonshot |
|  | 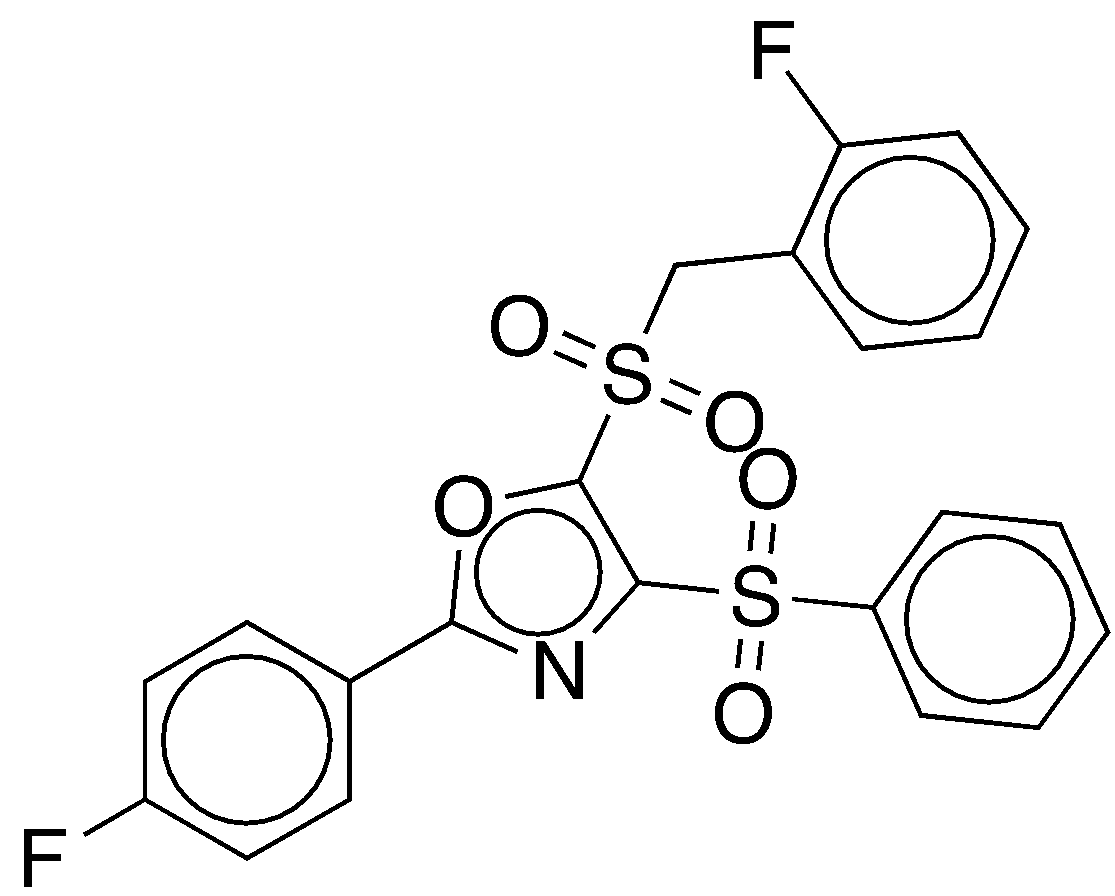 | | 0.38 | 9 | 475.491 | COVID Moonshot |
|  | 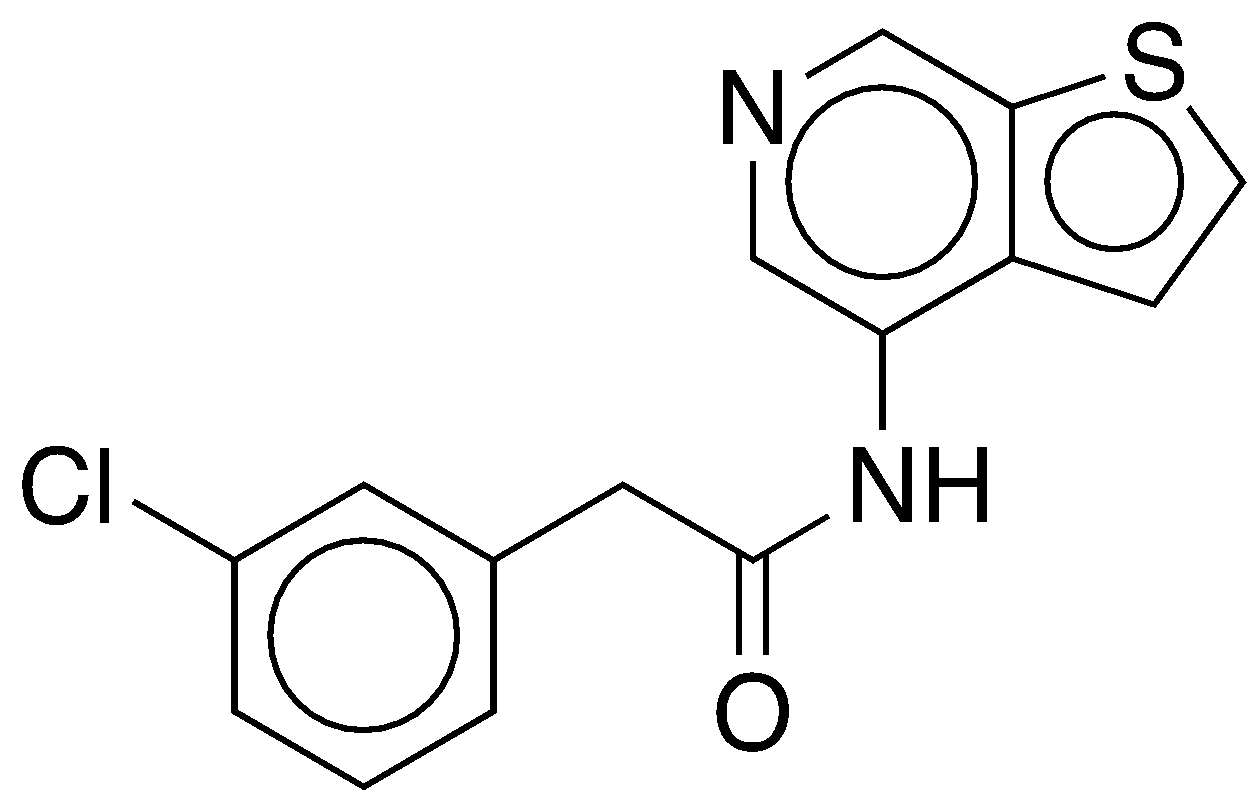 | | 0.41 | 5 | 302.784 | COVID Moonshot |
|  | 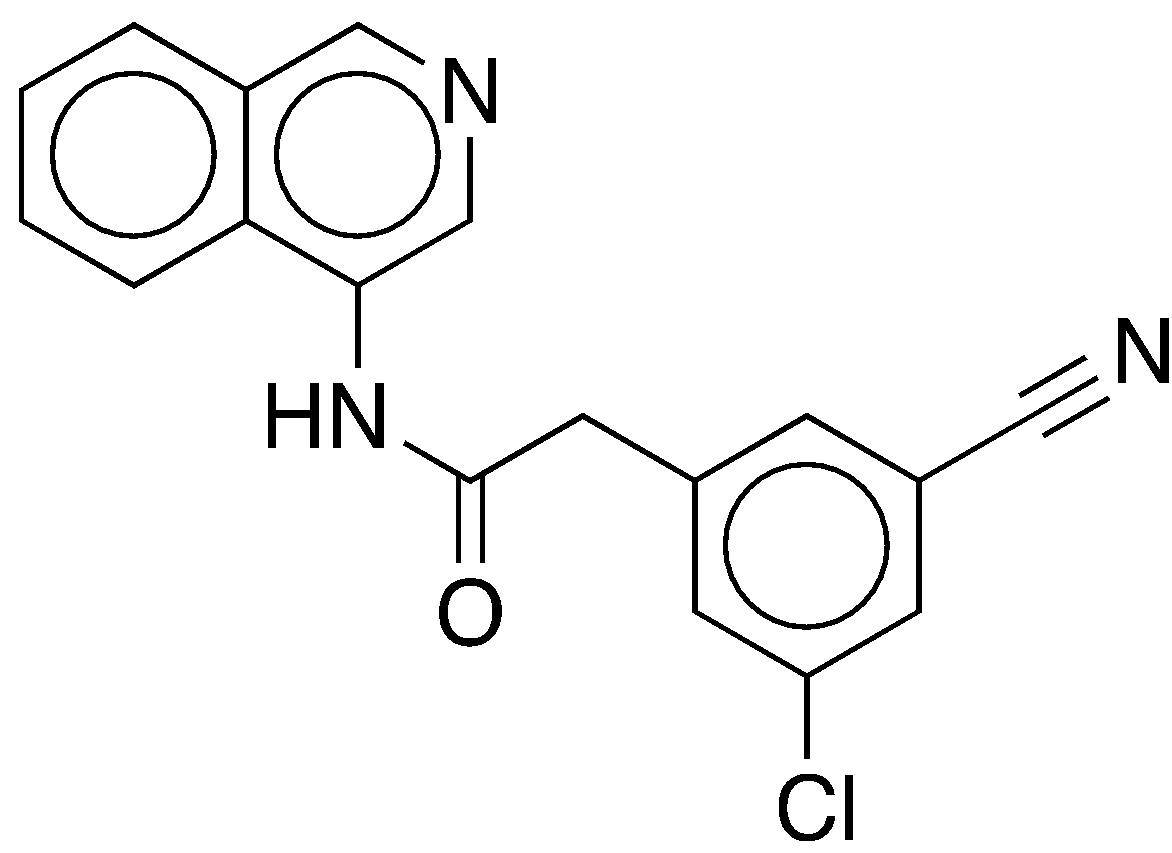 | | 0.43 | 5 | 321.766 | COVID Moonshot |
|  | 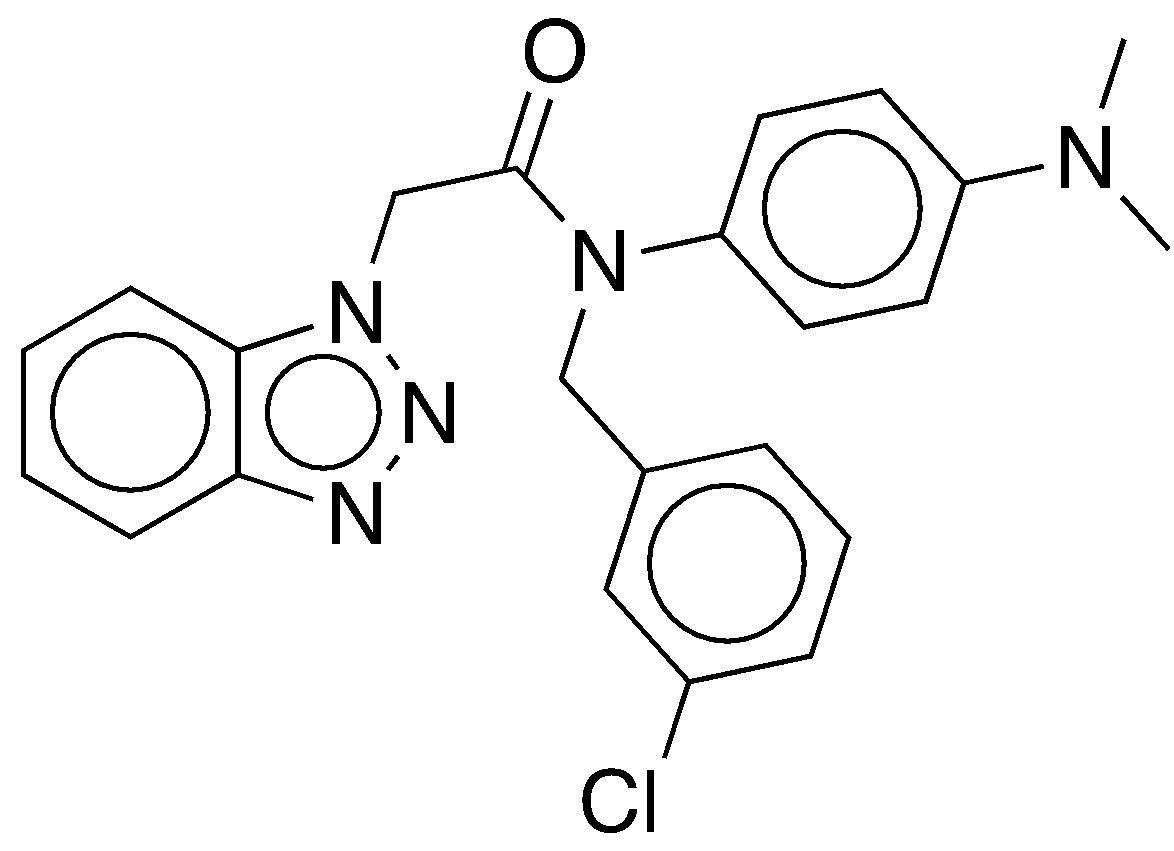 | | 0.49 | 8 | 419.915 | COVID Moonshot |
|  | 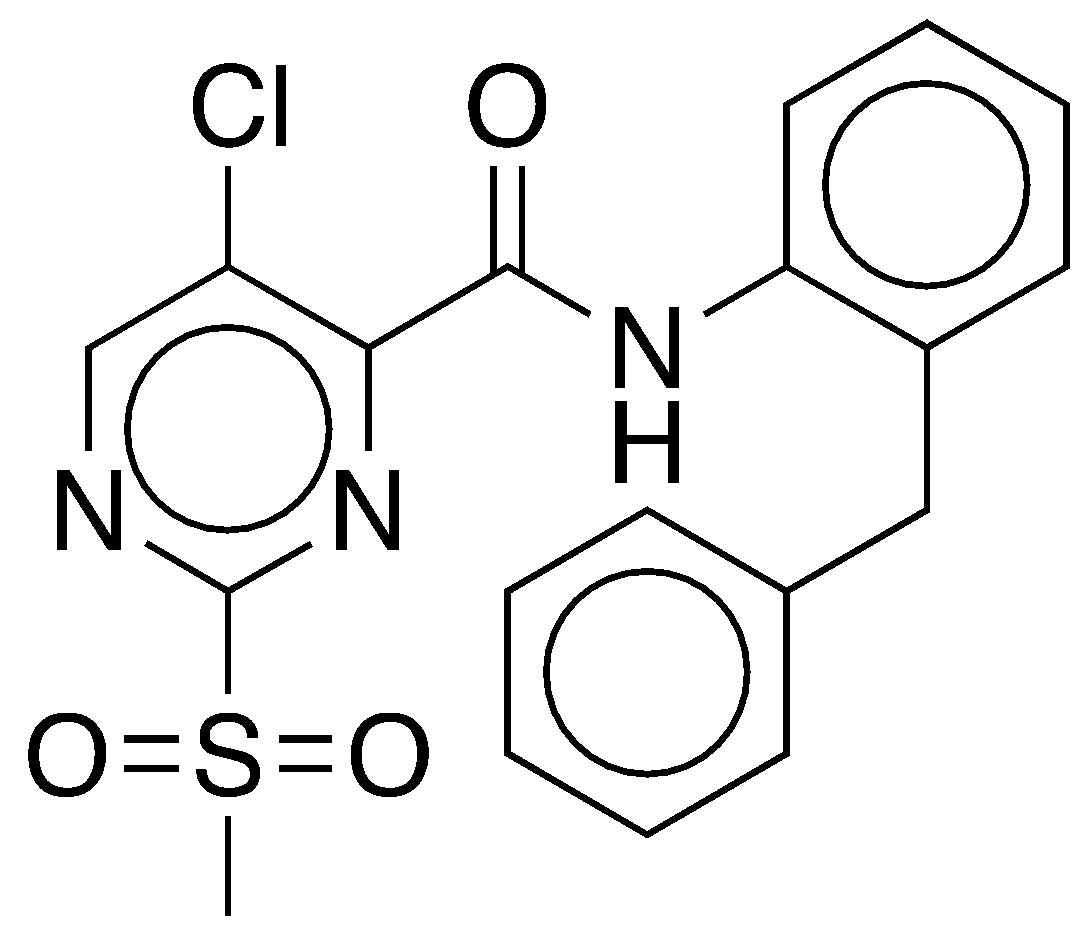 | | 0.5 | 2 | 401.873 | COVID Moonshot |
|  | 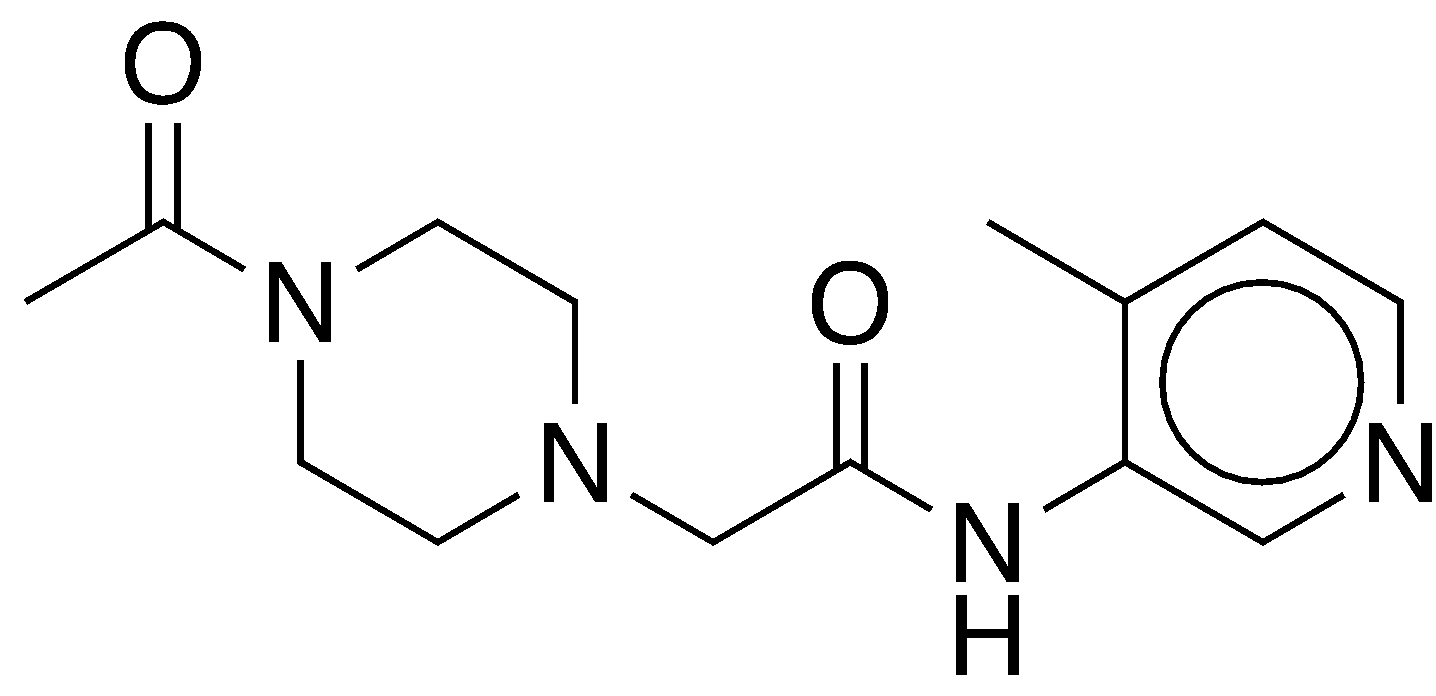 | | 0.63 | 10 | 276.339 | COVID Moonshot |
|  | 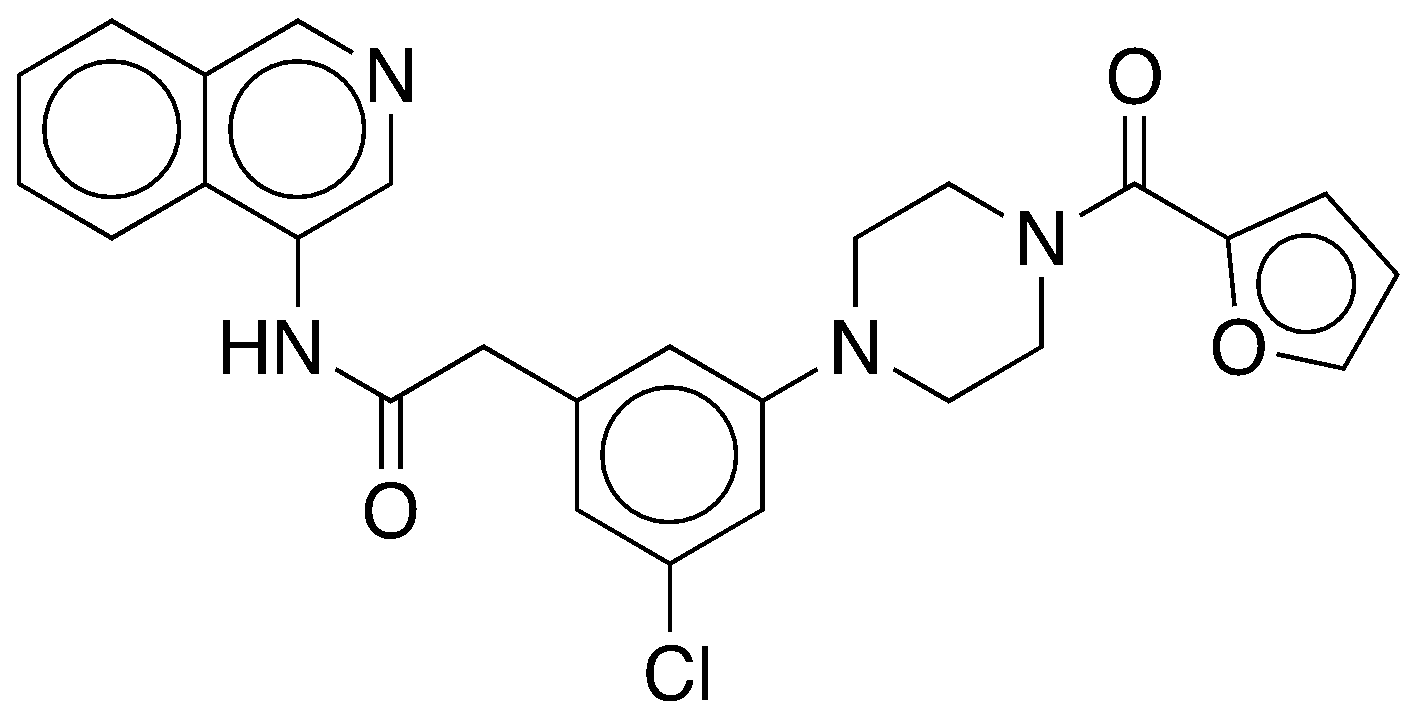 | | 0.63 | 10 | 474.947 | COVID Moonshot |
|  | 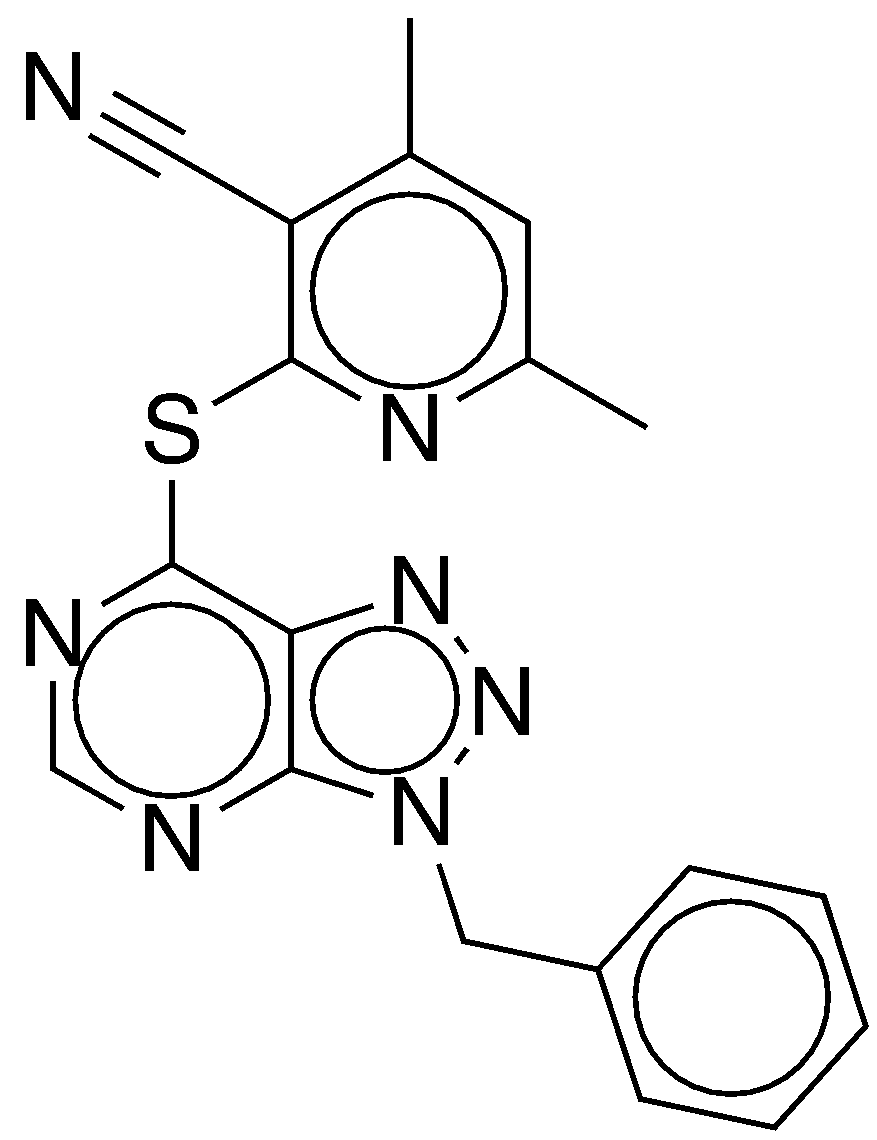 | | 0.63 | 11 | 373.443 | COVID Moonshot |
|  | 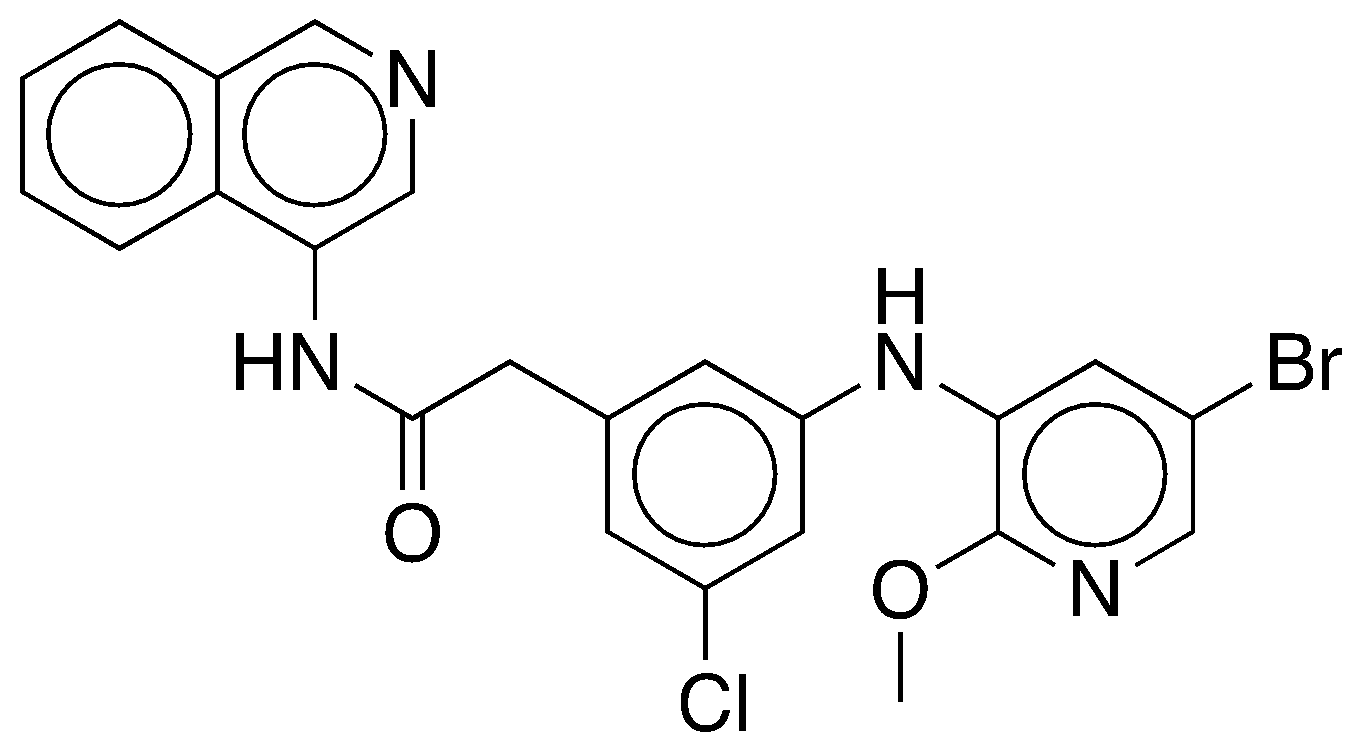 | | 0.68 | 5 | 497.779 | COVID Moonshot |
|  | 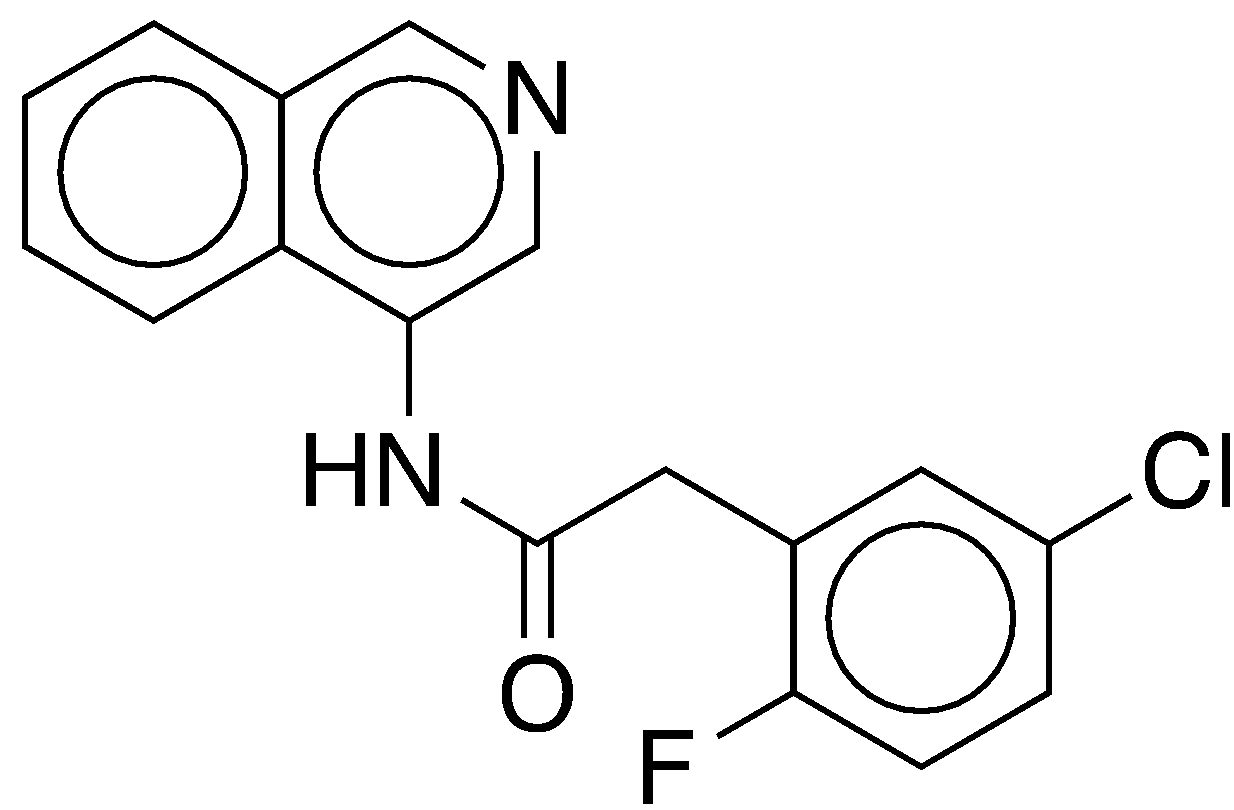 | | 0.69 | 5 | 314.746 | COVID Moonshot |
|  | 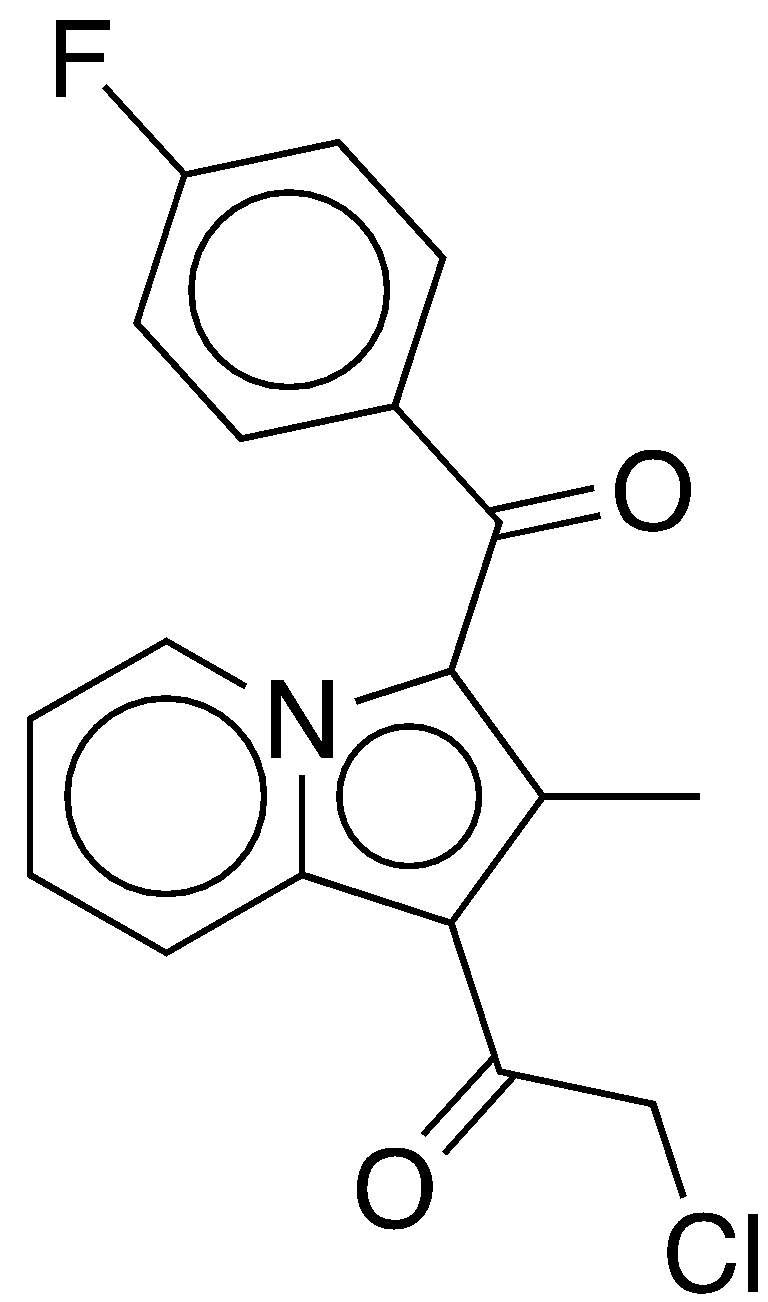 | | 0.7 | 12 | 329.757 | COVID Moonshot |
|  | 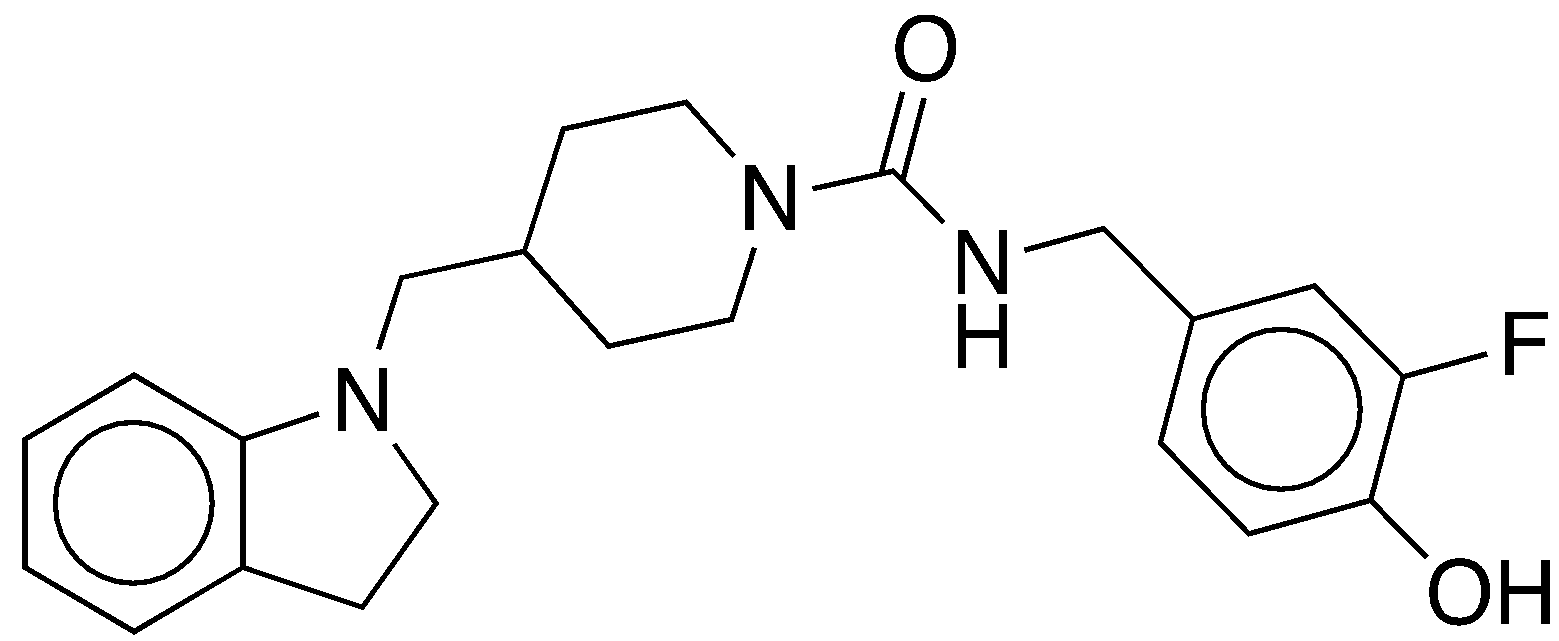 | | 0.73 | 13 | 383.465 | [9] |
|  | 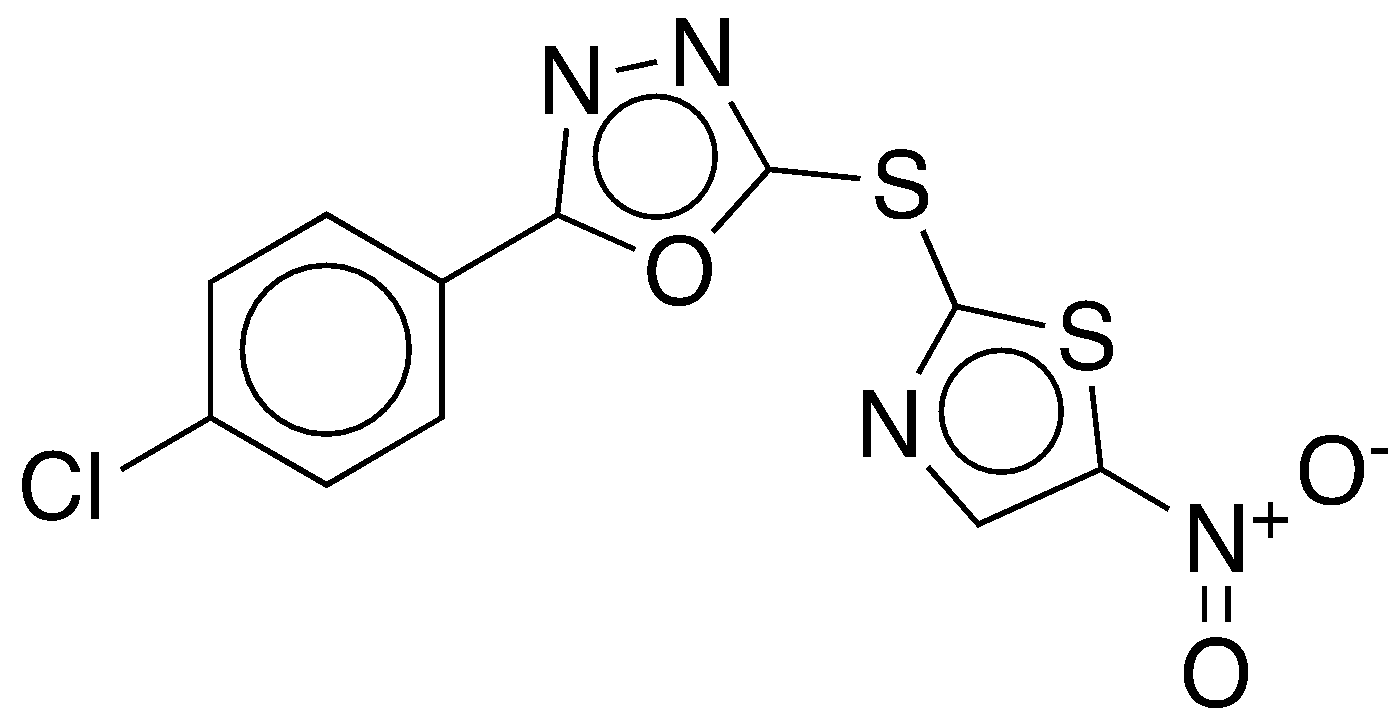 | | 0.88 | 14 | 340.771 | COVID Moonshot |
|  | 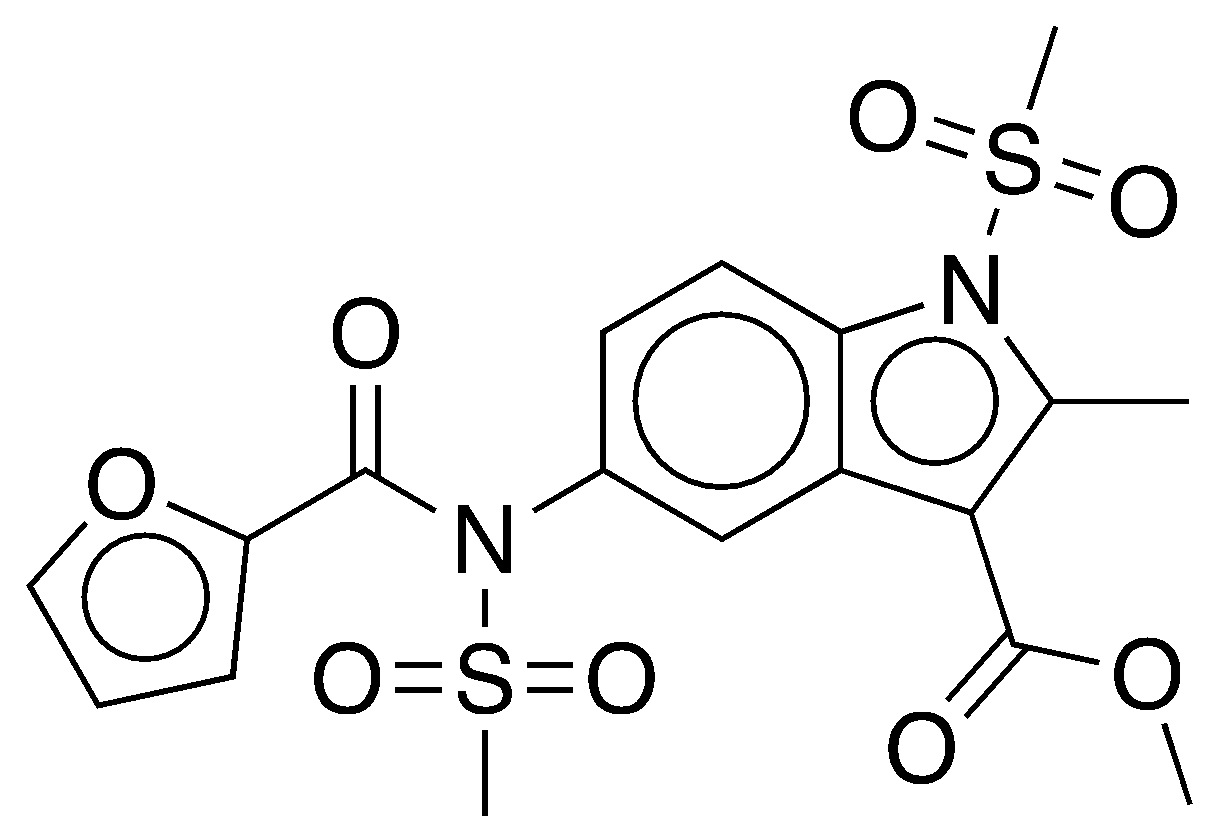 | | 0.93 | 8 | 454.479 | COVID Moonshot |
|  | 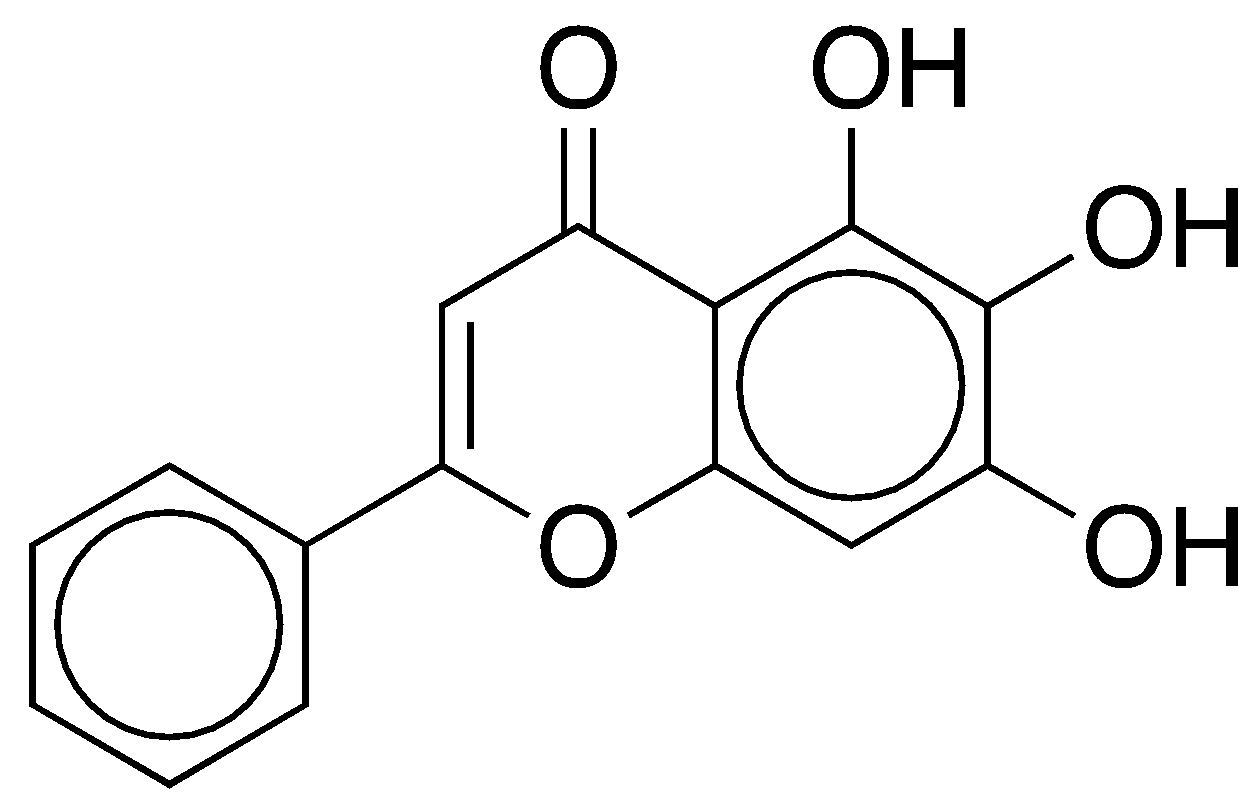 | | 0.94 | 18 | 269.231 | [10] |
|  | 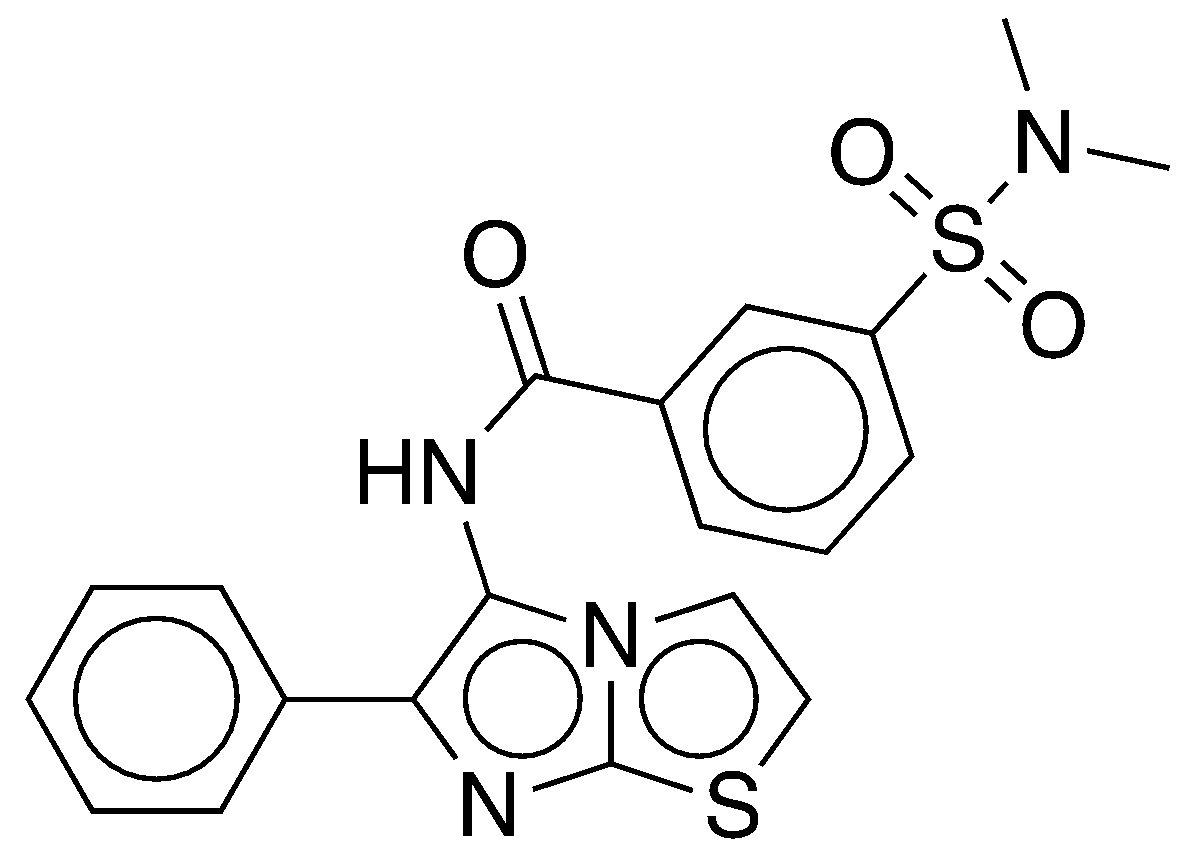 | | 0.97 | 15 | 426.52 | COVID Moonshot |
|  | 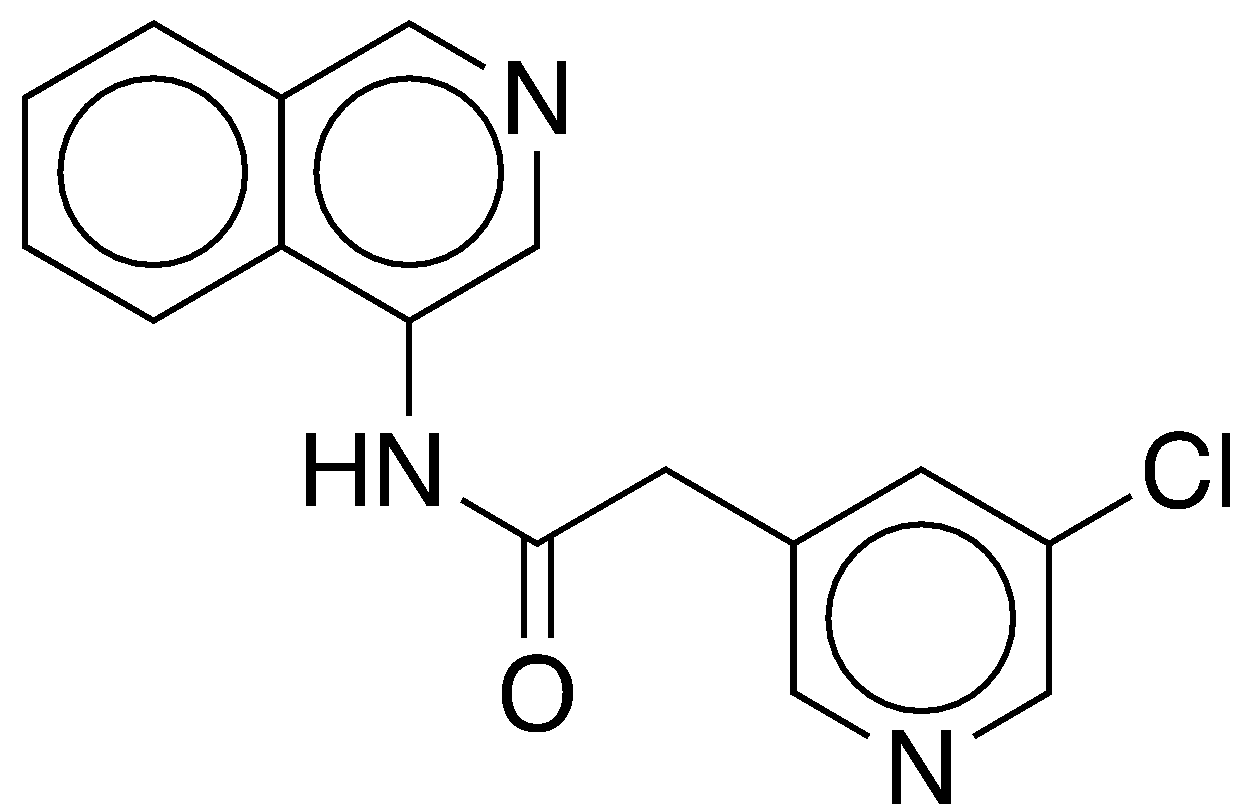 | | 0.99 | 5 | 297.744 | COVID Moonshot |
|  | 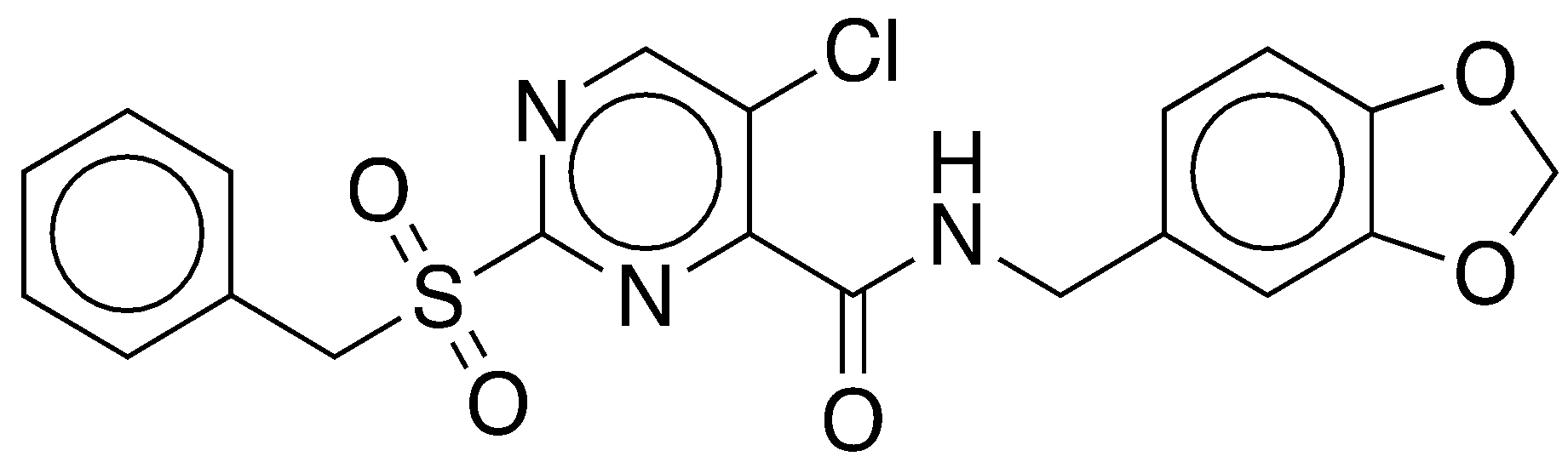 | | 1.39 | 16 | 445.882 | COVID Moonshot |
|  | 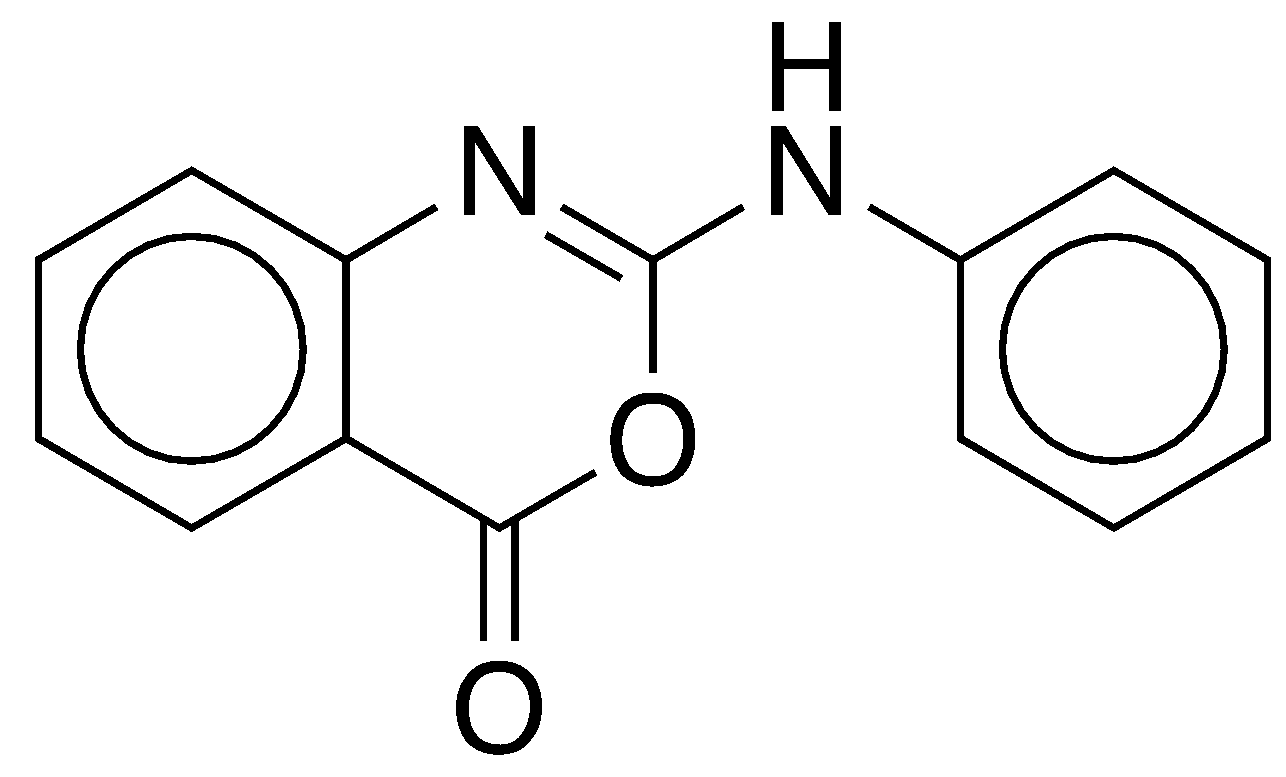 | | 1.57 | 9 | 238.245 | COVID Moonshot |
|  | 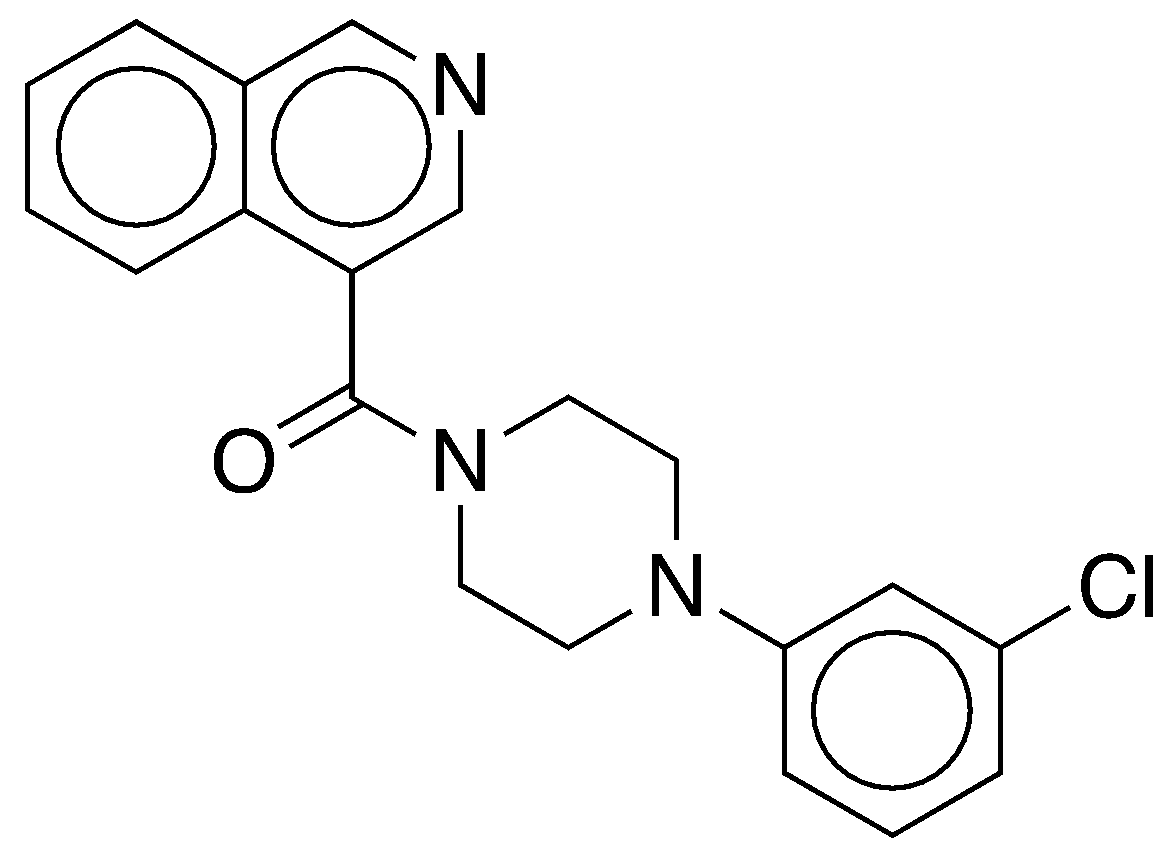 | | 1.6 | 19 | 351.836 | COVID Moonshot |
|  | 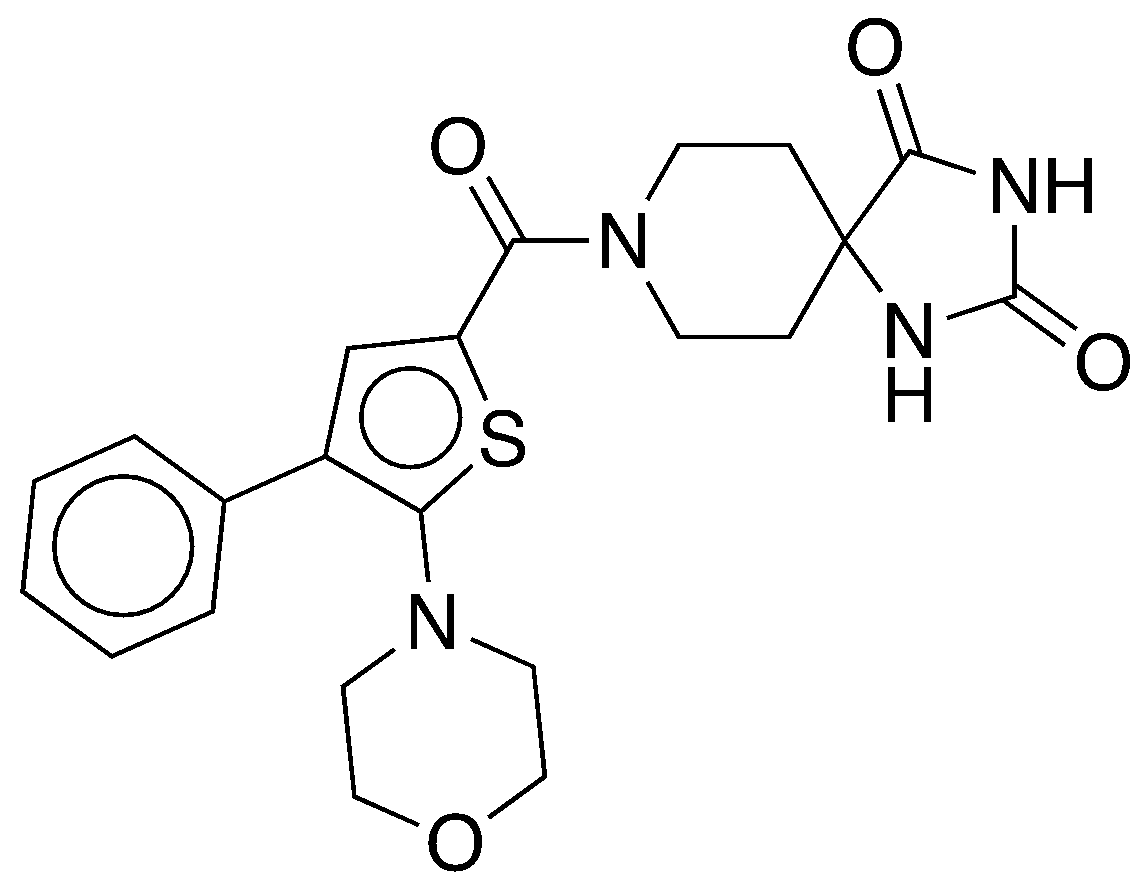 | | 1.72 | 17 | 440.523 | [9] |

**Table C.** The structures of the best enriched compounds against WTMpro and OMpro.

| **Structure** | **Compound ID** | **Vina Score**  **(Kcal/mol)** | **FRED Score** | **M. Wt.** | **Mpro Target** |
| --- | --- | --- | --- | --- | --- |
| 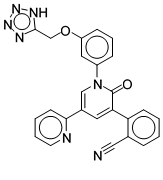 | **Compound 386** | -9.5  -10.7 | - | 447.45 | WT  (7L14)  (6LU7) |
| 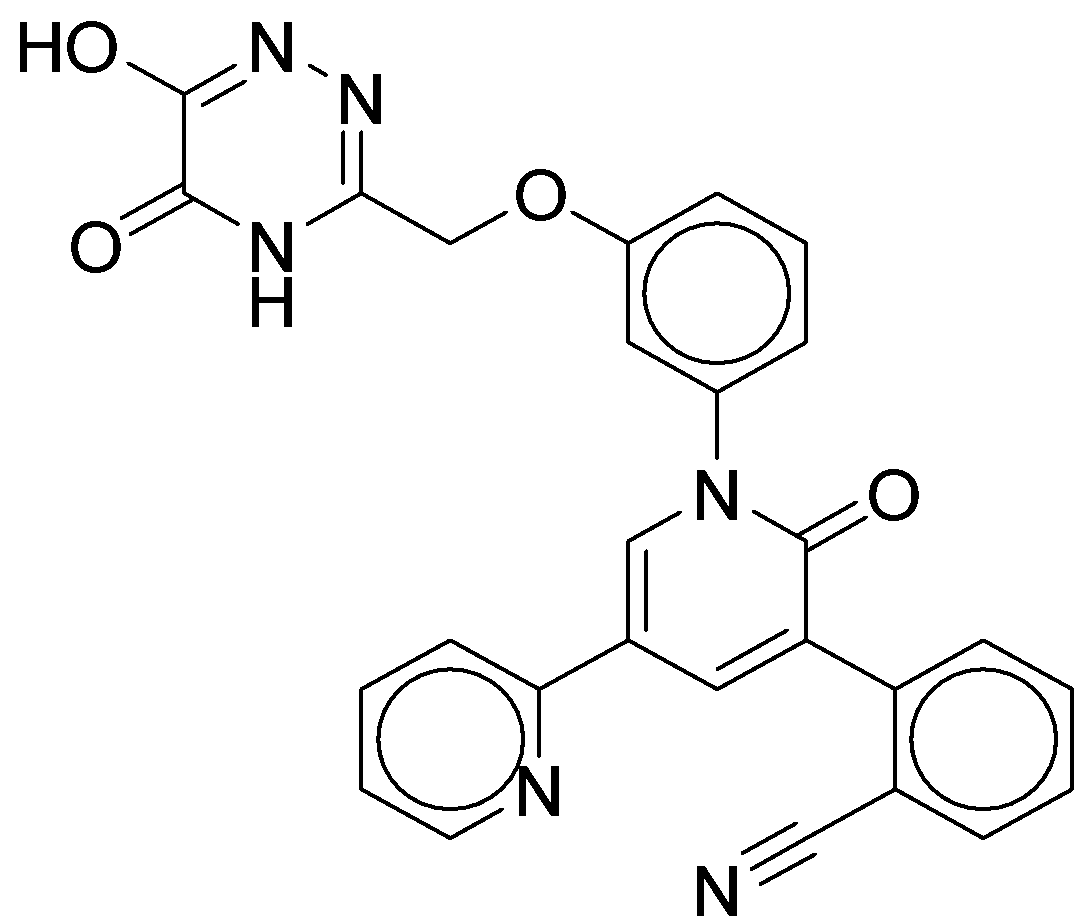 | **Compound 329** | -9.3  -10.2 | - | 490.47 | WT  (7L14)  (6LU7) |
| 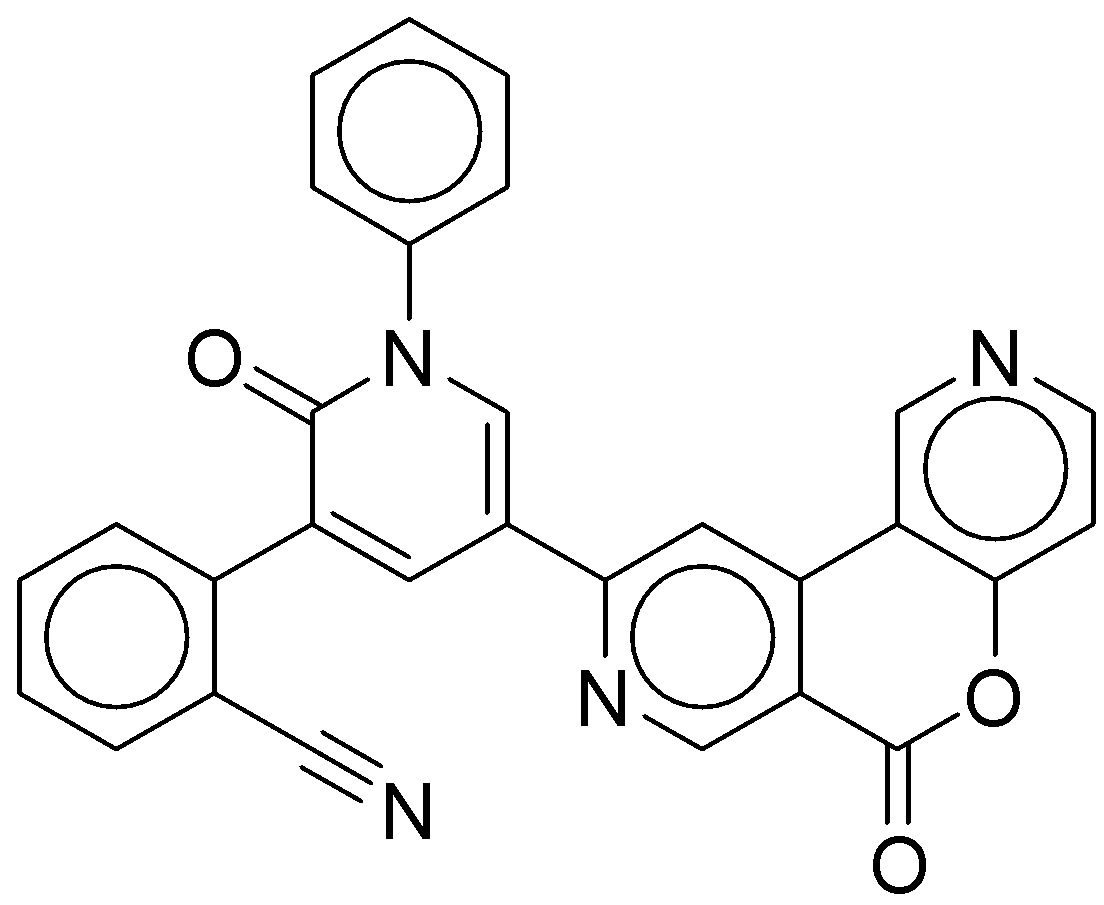 | **Compound 203** | -10.7  -10.9 | - | 468.46 | WT  (7L14)  (6LU7) |
| 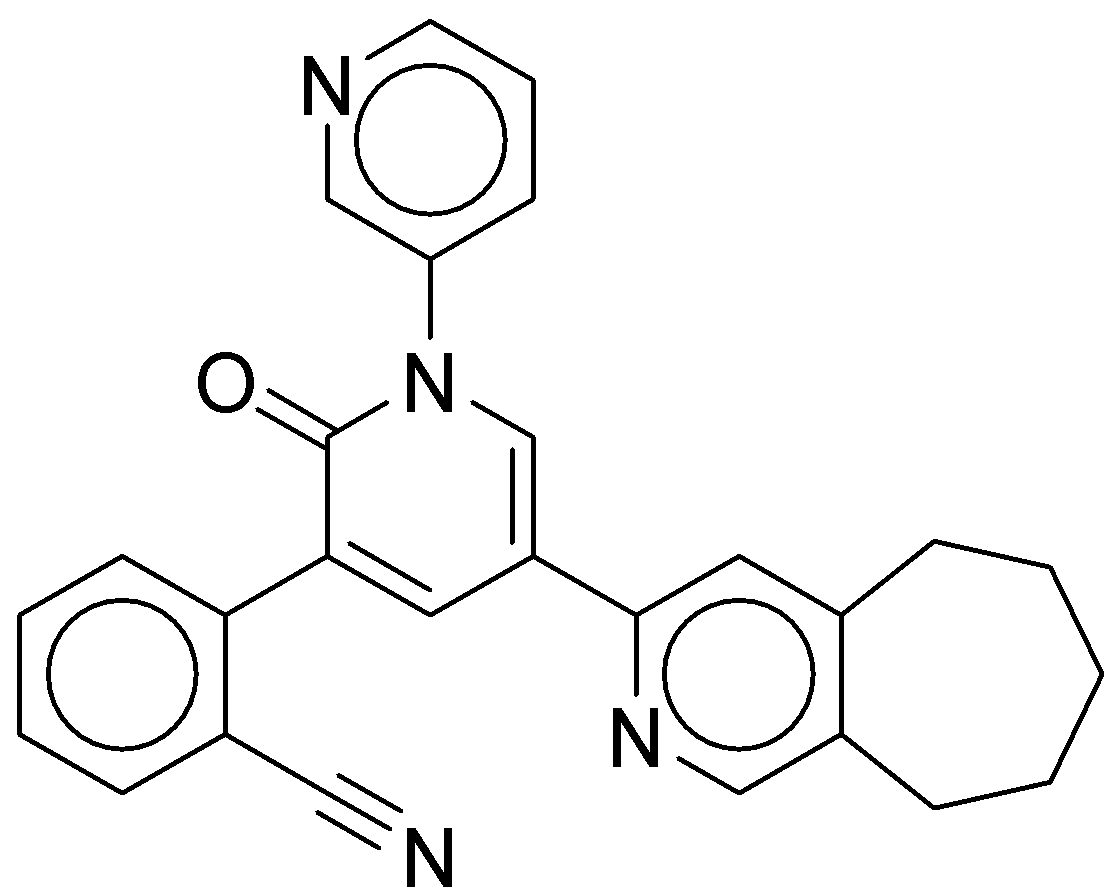 | **Compound 401** | -9.6 | -12.4 | 418.49 | Omicron  (P132H) |
| 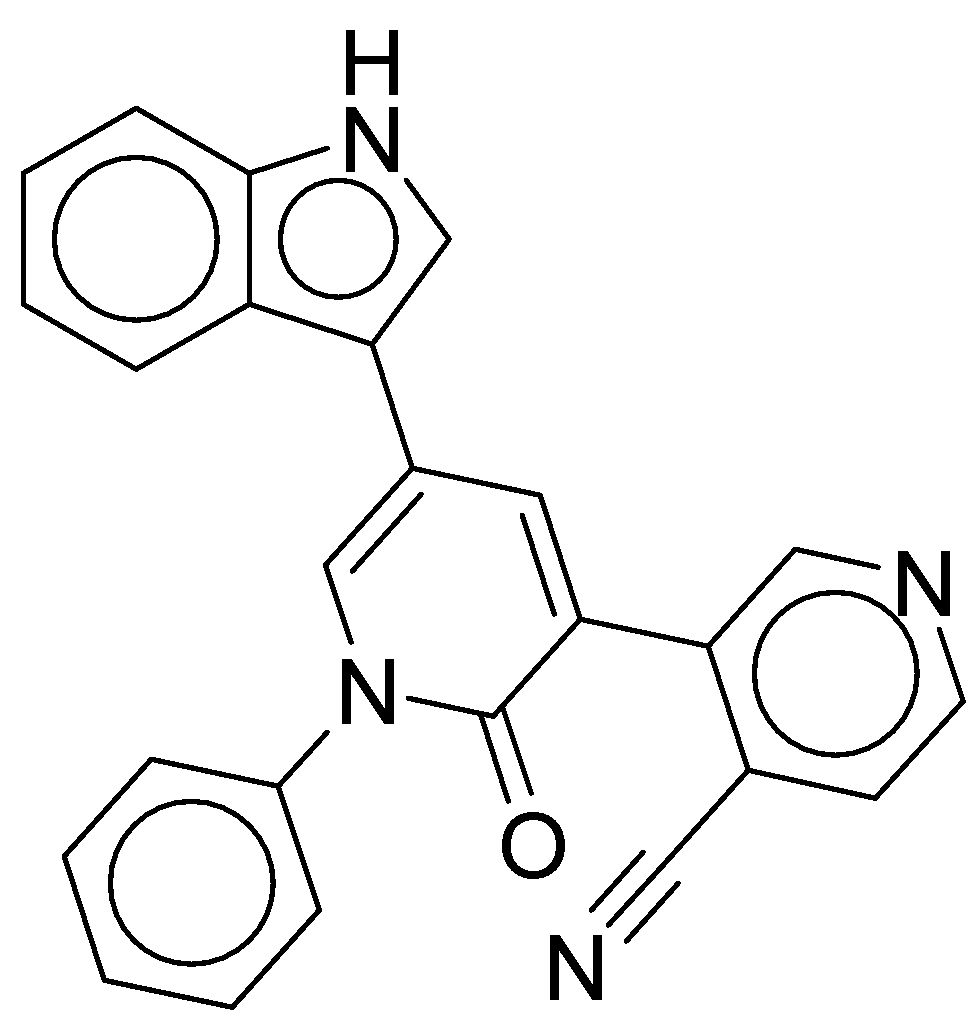 | **Compound 541** | -9.2 | -10.6 | 388.42 | Omicron  (P132H) |
| 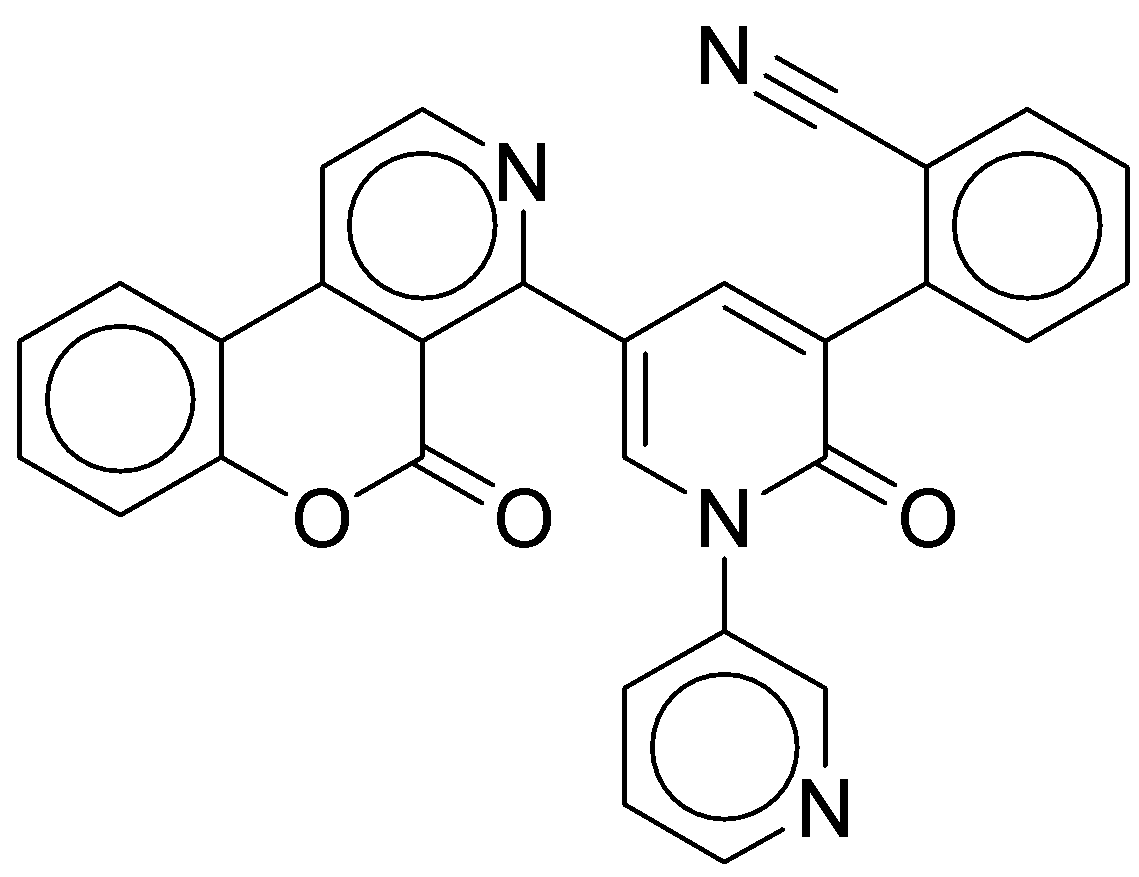 | **Compound 385** | -10.15 | -9.9 | 468.46 | Omicron  (P132H) |

**References**

1. Bussi, G., D. Donadio, and M. Parrinello, *Canonical sampling through velocity rescaling.* J Chem Phys, 2007. **126**(1): p. 014101.

2. Eslami, H., F. Mojahedi, and J. Moghadasi, *Molecular dynamics simulation with weak coupling to heat and material baths.* J Chem Phys, 2010. **133**(8): p. 084105.

3. Parrinello, M. and A.J.J.o.A.p. Rahman, *Polymorphic transitions in single crystals: A new molecular dynamics method.* 1981. **52**(12): p. 7182-7190.

4. Darden, T., D. York, and L.J.T.J.o.c.p. Pedersen, *Particle mesh Ewald: An N⋅ log (N) method for Ewald sums in large systems.* 1993. **98**(12): p. 10089-10092.

5. Hess, B., et al., *LINCS: a linear constraint solver for molecular simulations.* 1997. **18**(12): p. 1463-1472.

6. Huang, J. and A.D. MacKerell, Jr., *CHARMM36 all-atom additive protein force field: validation based on comparison to NMR data.* J Comput Chem, 2013. **34**(25): p. 2135-45.

7. Zoete, V., et al., *SwissParam: a fast force field generation tool for small organic molecules.* J Comput Chem, 2011. **32**(11): p. 2359-68.

8. Zhang, C.H., et al., *Potent Noncovalent Inhibitors of the Main Protease of SARS-CoV-2 from Molecular Sculpting of the Drug Perampanel Guided by Free Energy Perturbation Calculations.* ACS Cent Sci, 2021. **7**(3): p. 467-475.

9. Yang, J., et al., *Structure-Based Discovery of Novel Nonpeptide Inhibitors Targeting SARS-CoV-2 M(pro).* J Chem Inf Model, 2021. **61**(8): p. 3917-3926.

10. Su, H.X., et al., *Anti-SARS-CoV-2 activities in vitro of Shuanghuanglian preparations and bioactive ingredients.* Acta Pharmacol Sin, 2020. **41**(9): p. 1167-1177.
