# Supplementary material for: Evaluating the structure-based virtual screening performance of SARS-CoV-2 main protease: A benchmarking approach and a multistage screening example against the wild-type and Omicron variants
Source: PLoS One. 2025 Feb 19;20(2):e0318712. doi: 10.1371/journal.pone.0318712 (PMC11838920; doi:10.1371/journal.pone.0318712)
Supplement: Attachment — Submitted filename: Response-to-reviewers.docx [file pone.0318712.s002.docx]

**RESPONSE TO REVIEWERS’ COMMENTS**

**General Response:**

**We thank the reviewer for her/his time spent on recommending some improvements for our manuscript. This certainly helped us to enhance the quality of our manuscript. All changes are either blue-colored or in a “track-changes” mode in the manuscript.**

**Journal requirements:**

When submitting your revision, we need you to address these additional requirements.

**1. Please ensure that your manuscript meets PLOS ONE's style requirements, including those for file naming. The PLOS ONE style templates can be found at**

https://journals.plos.org/plosone/s/file?id=wjVg/PLOSOne_formatting_sample_main_body.pdf and

<https://journals.plos.org/plosone/s/file?id=ba62/PLOSOne_formatting_sample_title_authors_affiliations.pdf>

**Response: We revised the PLOS ONE’s style requirements and addressed all of them.**

**2. Please note that PLOS ONE has specific guidelines on code sharing for submissions in which author-generated code underpins the findings in the manuscript. In these cases, we expect all author-generated code to be made available without restrictions upon publication of the work. Please review our guidelines at https://journals.plos.org/plosone/s/materials-and-software-sharing#loc-sharing-code and ensure that your code is shared in a way that follows best practice and facilitates reproducibility and reuse.**

**Response: All findings are based on already published codes/methodologies. We cited them appropriately.**

**3. We noticed you have some minor occurrence of overlapping text with the following previous publication(s), which needs to be addressed:**

https://www.mdpi.com/1420-3049/28/3/1296

https://www.sciencedirect.com/science/article/pii/S0010482521002626?via%3Dihub

https://journals.plos.org/plosone/article?id=10.1371%2Fjournal.pone.0284301

In your revision ensure you cite all your sources (including your own works), and quote or rephrase any duplicated text outside the methods section. Further consideration is dependent on these concerns being addressed.

**Response: We addressed them and cited the references mentioned.**

**Reviewers’ comments:**

**Reviewer #1:** The work is quite interesting and the findings are well put together. The only concern here is the time scale of the MD simulation. I was wondering if there were any replicates carried out to validate the MD simulations, mostly for sanity check? If not, then 100 ns of MD with CHARMM is inadequate sampling in my opinion. This is also reflected in certain structural analysis, where the system stability is seen towards the end of the simulations. Longer simulation times might provide greater insight into such situations.

Along with the analysis performed, looking into non-covalent interaction energies to look into important residues that take part in binding and are important for stability might be useful. Using energy decomposition analysis for such is a good benchmark.

**Response: We thank the reviewer for raising this point.**

**Indeed, the main focus of our study is to provide the community with an evaluation/assessment of the virtual screening performance via in-depth benchmarking analysis against the highly represented protein target in literature for COVID-19, the Mpro. The molecular dynamics protocol is an auxiliary approach and example of how to in-silico confirm the binding event of the proposed hits in the binding site of both WTMpro and OMpro.**

**Furthermore, Mpro is extensively studied in literature and employing MD simulation for a 100 ns is a standard performance in many studies especially in the context of assessing ligand-protein stability, e.g., *PLOS ONE. 2023;18(4):e0284301. doi: 10.1371/journal.pone.0284301; and***

[**https://www.tandfonline.com/doi/full/10.1080/07391102.2022.2148000**](https://www.tandfonline.com/doi/full/10.1080/07391102.2022.2148000)

**Nonetheless, we already performed a pilot MD simulation for 200 ns during our experimental setup for the co-crystal ligand (WT – PDB ID: 7L14), as shown in Figure R below. The RMSD fluctuations (blue line) did not differ greatly after 100 ns of simulation time.**

**Based on all the previous, we decided to set the MD simulation time for 100 ns, and therefore, we kindly appreciate the reviewer’s understanding in this matter. We also provided extensive analyses of the MD trajectories including, MMGBSA free energy calculations, per-residue energy contribution, minimum distance analysis and providing different screenshots at different time points.**

**Figure R. For the WT (A) Ligand RMSD, (B) Protein RMSD and (C) hydrogen bonds analysis; blue color is for co-ligand, while red for compound 386 and green for compound 329, in all graphs.**

**Reviewer #2:** The manuscript is well structured and relevant to its field and can be proceeded to publication.
Authors cold just consider provide descriptions for both the bond angles and distances of their depicted ligand-target interaction patterns. Specially for Hydrogen bonding, this type of compound-protein polar interaction should be presented within hydrogen bond distances as well as bond angles since hydrogen bond depend on both. Authors should mention the Hydrogen bond angles as well as their distances, since the strength of hydrogen bonding is based on both parameters in a way to ensure the adequacy of optimum hydrogen bonding.

**Response: We thank the reviewer for his comment.**

**The H-bonding is depicted already in Figure 9 and 10 in the manuscript (H-bonding count). The native function of gromacs for H-bonding count considering both the distance and angle (please, see gromacs documentation). We quote the following from gromacs documentation: “A) Distance Criterion: The distance between the donor atom (typically a hydrogen bond donor such as an OH or NH group) and the acceptor atom must be less than or equal to *rHB*=0.35 nm. B) Angle Criterion: The angle formed by the hydrogen atom, the donor atom, and the acceptor atom must be less than or equal to *αHB*=30∘.”**

**Furthermore, based on the binding free energy (ΔG), in “*Binding free energy (ΔG) via MM/GBSA calculation*“ in the manuscript, the van der Waals energy was found to be the major contribution to the calculated ΔG for both WTMpro and OMpro complex systems. This minimizes the importance of providing more insights into some details for the H-bonding interactions.**

**Nonetheless, we already provided detailed distance analysis of the key H-bonding interactions for the WTMpro in the section “ *Minimum Distance analysis”.***

**Thank you,**
